# Persistent delay in maturation of the developing gut microbiota in infants with cystic fibrosis

**DOI:** 10.1128/mbio.03420-24

**Published:** 2025-02-13

**Authors:** Adrian J. Verster, Paige Salerno, Rebecca Valls, Kaitlyn Barrack, Courtney E. Price, Emily A. McClure, Juliette C. Madan, George A. O’Toole, Julie L. Sanville, Benjamin D. Ross

**Affiliations:** 1 Department of Microbiology and Immunology, Geisel School of Medicine, Dartmouth College, Hanover, New Hampshire, USA; 2 Department of Pediatrics, Geisel School of Medicine, Dartmouth College, Hanover, New Hampshire, USA; 3 Department of Epidemiology, Geisel School of Medicine, Dartmouth College, Hanover, New Hampshire, USA; Georgia Institute of Technology, Atlanta, Georgia, USA; Georgia Institute of Technology, Atlanta, Georgia, USA

**Keywords:** metagenomics, gut microbiota, cystic fibrosis

## Abstract

The healthy human infant gut microbiome undergoes stereotypical changes in taxonomic composition between birth and maturation to an adult-like stable state. During this time, extensive communication between microbiota and the host immune system contributes to health status later in life. Although there are many reported associations between microbiota compositional alterations and disease in adults, less is known about how microbiome development is altered in pediatric diseases. One pediatric disease linked to altered gut microbiota composition is cystic fibrosis (CF), a multi-organ genetic disease involving impaired chloride secretion across epithelia and heightened inflammation both in the gut and at other body sites. Here, we use shotgun metagenomics to profile the strain-level composition and developmental dynamics of the infant fecal microbiota from several CF and non-CF longitudinal cohorts spanning from birth to greater than 36 months of life. We identify a set of keystone species that define microbiota development in early life in non-CF infants but are missing or decreased in relative abundance in infants with CF, resulting in a delayed pattern of microbiota maturation, persistent entrenchment in a transitional developmental phase, and subsequent failure to attain an adult-like stable microbiota. Delayed maturation is strongly associated with cumulative antibiotic treatments, and we also detect the increased relative abundance of oral-derived bacteria and higher levels of fungi in infants with CF, features that are associated with decreased gut bacterial density. These findings suggest the potential for future directed therapies targeted at overcoming developmental delays in microbiota maturation for infants with CF.

**IMPORTANCE:**

The human gastrointestinal tract harbors a diversity of microbes that colonize upon birth and collectively contribute to host health throughout life. Infants with the disease cystic fibrosis (CF) harbor altered gut microbiota compared to non-CF counterparts, with lower levels of beneficial bacteria. How this altered population is established in infants with CF and how it develops over the first years of life is not well understood. By leveraging multiple large non-CF infant fecal metagenomic data sets and samples from a CF cohort collected prior to highly effective modulator therapy, we define microbiome maturation in infants up to 3 years of age. Our findings identify conserved age-diagnostic species in the non-CF infant microbiome that are diminished in abundance in CF counterparts that instead exhibit an enrichment of oral-derived bacteria and fungi associated with antibiotic exposure. Together, our study builds toward microbiota-targeted therapy to restore healthy microbiota dynamics in infants with CF.

## INTRODUCTION

The human gut microbiota is composed of hundreds of diverse microbial species that contribute to human health through effects on immune, metabolic, and physiological function (1). Alterations in the composition of the gut microbiota are linked to a variety of human diseases, and it is of great basic and translational interest to understand how such altered states arise and by what mechanism they influence human health. The assembly of the neonatal gut microbiota begins at birth and proceeds to undergo dynamic and stereotypical developmental transitions through early life, defined by compositional shifts in the diversity, prevalence, and relative abundance of specific microbial taxa (2, 3). Microbiota compositional dynamics during this period are shaped by intrinsic forces, such as colonization priority effects and competition between co-resident bacteria, as well as external factors, including delivery mode, breastfeeding status, and antibiotic exposure (4). Between 3 to 5 years of age, these dynamics stabilize into an adult-like microbiome composition (5, 6). Disruptions to this process of gut microbiota maturation can be associated with poor health. For example, children with acute malnutrition exhibit aberrant gut microbiome maturation associated with deficits in growth, as well as altered metabolic, immune, and neurological development (7). Microbiota-directed dietary interventions are correlated with improvements to child growth and development, demonstrating the interrelation between microbiota development and health (8, 9). In mice, disruptions to microbiome maturation in early life result in heightened susceptibility to inflammation through negative impacts on immune development (10–15). Together, these observations provide evidence that gut microbiota maturation in early life contributes to healthy development.

Cystic fibrosis (CF) is a genetic disease defined by mutations in the gene encoding the cystic fibrosis transmembrane conductance regulator CFTR, which affects chloride ion transport across cell membranes in multiple organs (16). People with CF experience a range of gastrointestinal complications, including chronic inflammation that resembles other intestinal inflammatory conditions, and a systemic hyper-inflammatory state is present at birth, even in the absence of bacterial infections of the lung, the organ most associated with morbidity and mortality (17–19). Infants and young children with CF are dramatically affected and typically require intensive medical attention from birth, with nutritional and growth deficits due to pancreatic insufficiency and progressive lung disease (20, 21). Dramatic alteration of the gut microbiota composition is a hallmark of both infants and adults with CF, correlating with linear growth inhibition and increased fecal fat content and markers of intestinal inflammation (22–28). Notable compositional differences in the microbiota found across studies between control and CF individuals from both infant and adult cohorts include increased Proteobacteria and diminished levels of Bacteroidetes, which manifest following birth and do not resolve (22, 23, 26, 27, 29). People with CF are often repeatedly exposed to antibiotics, pancreatic enzyme replacement therapy, proton pump inhibitor therapy, and altered diet, all factors which can plausibly alter the microbiome independently (30, 31). However, carefully controlled gnotobiotic mouse studies in which specific pathogen free fecal material was transplanted into germ-free CF or non-CF mice revealed that CFTR dysfunction alone is sufficient to drive significant differences in the microbiota (32). Regardless of the underlying mechanism(s), there is accumulating evidence that alterations in the gut microbiota in CF are linked to disease at distal sites (33). For example, reduced fecal levels of short chain fatty acid (SCFA) producing gut microbes are separately associated with diminished growth and inflammation (26, 34). Communication between the gut microbiota and the lung may also influence disease progression in CF through the systemic diffusion (or lack of diffusion) of microbially produced metabolites or through influencing inflammation or immune cells (33).

The extent to which microbiota maturation defects in CF persist past 12 months is not fully understood. To better define microbiota longitudinal development in CF, we performed metagenomic shotgun sequencing on fecal samples from a prospective cohort of 40 infants with CF to better define the taxonomic and functional development of the gut microbiome over the first 3 years of life. Here, through a comparison with a large number of non-CF infant samples from published metagenomic data sets, we identify a delay in microbiome developmental maturation in CF that persists past 3 years of age and reveal strain-level insight into alterations in the CF gut microbiota that could be targets for therapeutic restoration.

## RESULTS

### Altered taxonomic composition of the fecal microbiota of infants with CF

**Fig 1:**
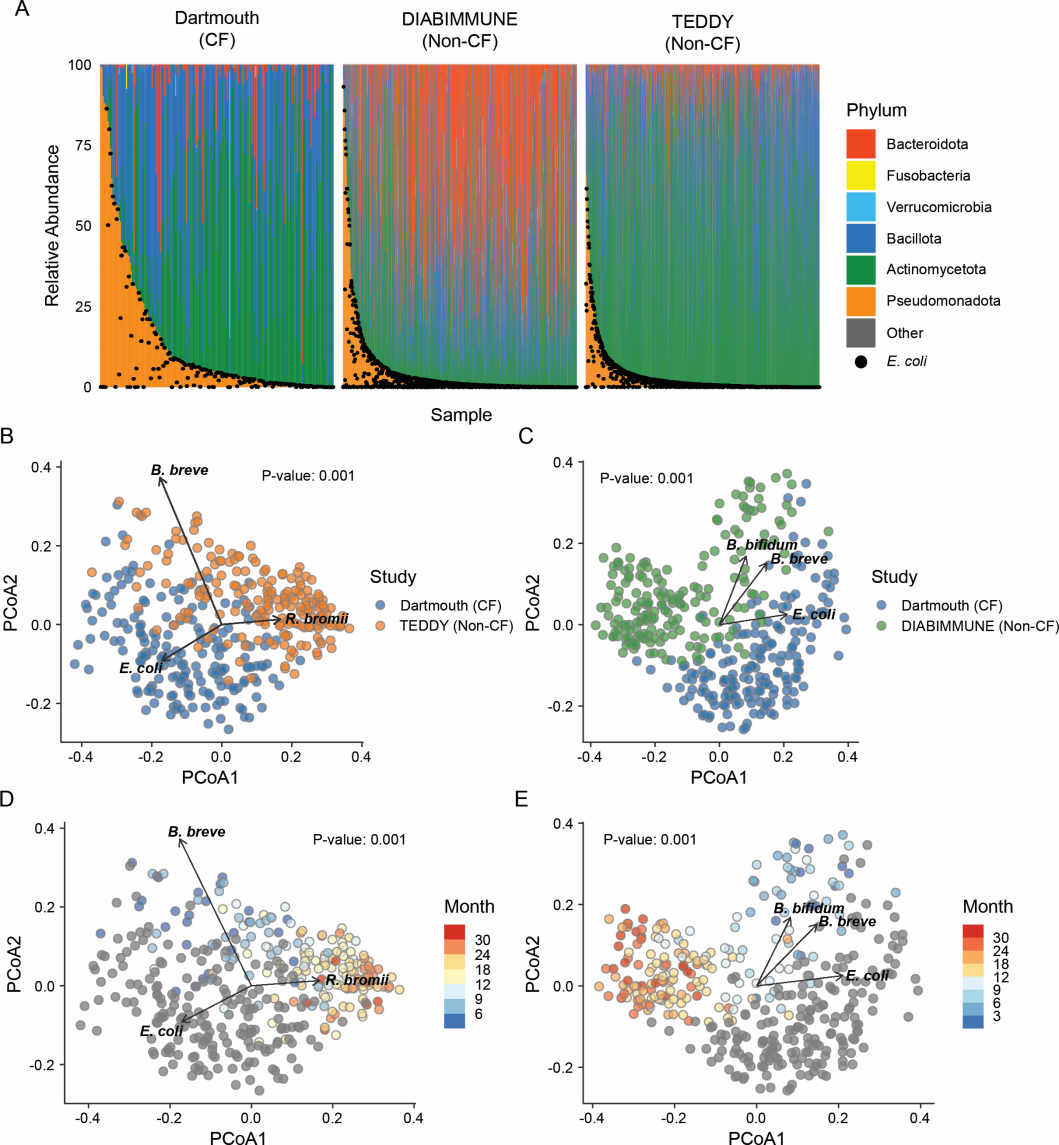
Alteration of gut microbiota composition in infants with CF. (**A**) Phylum level abundance of infants in the Dartmouth (left), DIABIMMUNE (center), and TEDDY (right) cohorts from ages 0 to 36 months. Samples are organized by relative abundance of proteobacteria. Black dots represent the *E. coli* relative abundance for each sample (*n* = 2,589). (**B and C**) PCoA of Dartmouth (blue) and TEDDY (B, orange) or DIABIMMUNE (C, green) samples from ages 0 to 36 months (unweighted UniFrac, PERMANOVA). A subset of non-CF samples in both cases was randomly selected to match the number of samples in the Dartmouth cohort (*n* = 189). Vectors indicate the top three species responsible for the separation of CF samples from each of the two non-CF data sets based on Euclidean distance calculations. (**D and E**) PCoA of Dartmouth and TEDDY (**D**) or DIABIMMUNE (**E**) samples from ages 0 to 36 months (unweighted UniFrac, PERMANOVA). Control samples were binned and colored by age with Dartmouth CF samples colored gray. A subset of non-CF samples in both cases was randomly selected to match the number of samples in the Dartmouth cohort (*n* = 189). Vectors indicate the top three species responsible for the separation of the two data sets based on Euclidean distance calculations.

We first investigated the taxonomic composition and developmental dynamics of the fecal microbiota of infants with CF. To accomplish this, we performed shotgun metagenomic sequencing on longitudinally collected fecal samples from a retrospective cohort of 40 infants with CF from northern New England from birth through the first 3 years of life (hereafter referred to as the Dartmouth cohort, Tables S1 and S2). This cohort was not exposed to highly effective modulator therapy and was balanced in terms of sex and birth mode (Tables S1 and S2). Most individuals possessed at least one F508del mutation. A subset of the samples from this cohort was previously analyzed by 16S rRNA gene amplicon sequencing (22, 35). To generalize our findings, we also acquired a large set of previously published metagenomics shotgun sequencing data sets derived from CF and non-CF infants comprising 3,863 samples from North America and northern Europe (Tables S3 and S4) (3, 6, 26, 36–38). Taxonomic profiling confirmed previous findings that infants with CF possess dramatic phylum-level microbiota alterations compared with non-CF counterparts, with notable deficits in the abundance of Bacteroidota and elevated levels of Pseudomonadota (formerly Proteobacteria) (Fig. 1A; Fig. S1; Table S5) (26). Species-level tests of microbiota compositional differences in CF revealed significant separation from microbiota of non-CF infants along a gradient of the relative abundance of *Escherichia coli*, *Ruminococcus bromii*, and *Bifidobacterium breve* (CF vs. TEDDY, unweighted UniFrac, *P* < 0.001, PCoA; Fig. 1B) and *E. coli*, *B. breve*, and *B. bifidum* (CF vs. DIABIMMUNE, unweighted UniFrac, *P* < 0.001, PCoA; Fig. 1C). Differences in microbiota composition between CF and non-CF samples are pronounced across all age bins (CF vs. non-CF, unweighted UniFrac, *P* < 0.001, PCoA; Fig. 1D and E).

Restricting our analysis to only those samples collected up to 12 months of age, we compared microbiota composition between the two CF data sets (Dartmouth and Hayden et al.). We found that CF microbiomes in the first year of life share similar taxonomic profiles at the phylum level (Fig. S2A). Notably, we found that clustering of CF samples in the absence of non-CF samples revealed separation into two distinct groups defined by the presence or absence of detected *Bacteroides* (unweighted UniFrac, *P* < 0.001, PCoA; Fig. S2B and C). This finding is reminiscent of previously reported microbiome states observed in a non-CF infant cohort (3), and we similarly find that this *Bacteroides*-deficient microbiota signature was not differentiated by any measured clinical parameter, including breastfeeding status, fecal fat, proton pump inhibitor usage, antibiotic treatment, or fecal calprotectin levels (data not shown). Despite this signature of within-CF compositional divergence in the first year of life, CF infant samples from both data sets cluster separately from non-CF counterparts, driven largely by the relative abundance of *Bifidobacteria bifidum*, *Phoecicola vulgatus*, and *E. coli* (unweighted UniFrac, PCoA; Fig. S2D). Taken together, these data demonstrate that the species-level composition of the gut microbiota of infants with CF differs dramatically from that of non-CF infants.

### Microbiome dynamics in early life

The healthy infant microbiome exhibits highly stereotypical dynamic changes in taxonomic composition over the first 3 to 5 years of life, with the most dramatic changes occurring in the first 12 months (2, 3, 5, 6). Infants with CF were previously reported to possess delays in gut microbiota development in the several years of life (26, 35, 39). We sought to replicate these findings in a different cohort. Toward this end, we first assessed family-level taxonomic profiles in the non-CF infant data and found stereotypical temporal patterns with decreasing levels of Enterobacteriaceae over the first 12 months of age corresponding with the increasing abundance of Bacteroidaceae and Lachnospiraceae (Fig. S3A). Following the first 12 months, non-CF microbiota continue to exhibit expansion of Bacteroidaceae and Ruminococcaceae populations. In contrast, this pattern was not observed for infants with CF, who instead possess high levels of Enterobacteriaceae in the first 12 months and persistently elevated levels of Bifidobacteriaceae at the expense of other families (Fig. S3A). We additionally examined changes in alpha diversity over time. In non-CF infants, as previously reported, fecal microbiome alpha diversity increases steadily in the first year before stabilizing past 24 months of age (5, 6). Previous reports indicated that infants with CF exhibit significantly lower alpha diversity compared to controls (22, 26, 29). However, in contrast with these prior studies, we found minimal significant differences in Shannon diversity between non-CF and CF cohorts, except when comparisons included the relatively small non-CF cohort examined in Hayden et al. (Fig. S3B through D, Wilcoxon ranked sum test) (26). Notably, from 0 to 6 months of age, the non-CF infants from the TEDDY study exhibited lower Shannon diversity than CF cohorts, perhaps due to the very high relative abundances of Actinomycetota observed in this cohort (Fig. 1A; Fig. S3D) (6, 37).

### Persistent delay in microbiota maturation in infants with CF

**Fig 2:**
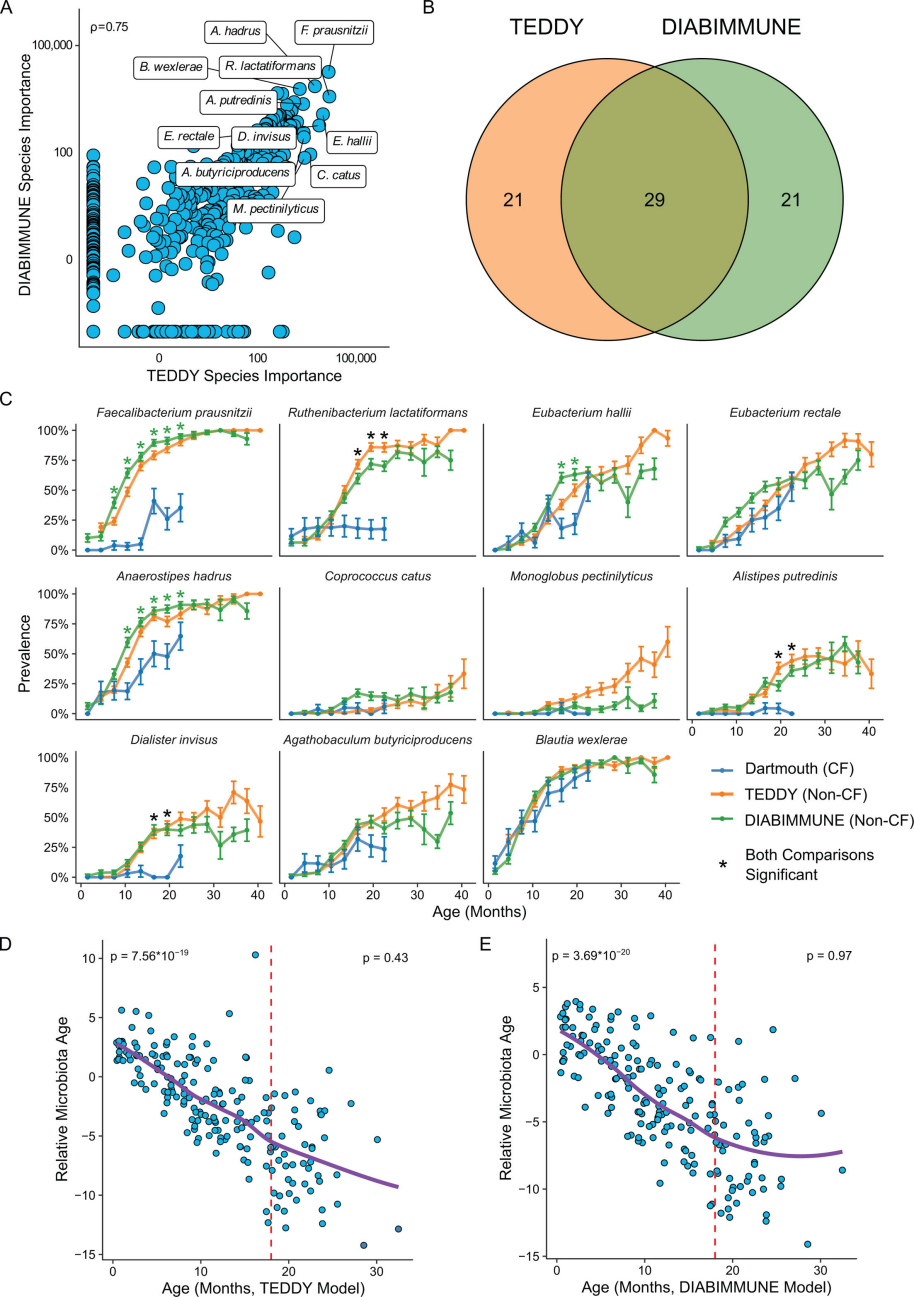
Persistent decline in microbiota relative age in infants with CF infants. (**A**) Correlation plot showing species importance values from microbiota age models trained on TEDDY and DIABIMMUNE data sets. Datapoints represent individual species, and the top 11 species are labeled. ρ = 0.75, Spearman correlation (*n* = 2,400). (**B**) Venn diagram showing the overlap of the top 50 most important species between TEDDY and DIABIMMUNE models (*n* = 2,400). (**C**) Prevalence plots for each of the top 11 species in Dartmouth (blue), DIABIMMUNE (green), and TEDDY (orange) in the first 40 months. Stars indicate statistical significance for comparisons between Dartmouth and TEDDY (orange), Dartmouth and DIABIMMUNE (green), or both (black). *P < 0.001, Fisher’s exact test (*n* = 2,590). (**D**) Relative microbiota age progression in the Dartmouth cohort when compared to the TEDDY age model. Red dotted line (18 months) signifies the time point at which the slope of the line is no longer significant. *P* value on the upper left corner is slope prior to 18 months; *P* value on the upper right is slope after 18 months (*n* = 190). (**E**) Relative microbiota age progression in the Dartmouth cohort when compared to the DIABIMMUNE age model. Red dotted line (18 months) signifies the time point at which the slope of the line is no longer significant. *P* value on the upper left corner is slope prior to 18 months; *P* value on the upper right is slope after 18 months (*n* = 190).

To gain deeper insight into the nature of the altered developmental dynamics of the CF infant gut microbiota, we generated regularized random forest models as have been used previously in building models of relative microbiota age (7, 26). The models were trained on species-level longitudinal taxonomic profiles derived from non-CF infant gut microbiome samples from the TEDDY (*n* = 1,246) and DIABIMMUNE (*n* = 1,154) studies. For each model generated from the two non-CF infant data sets, we modeled the chronological age as a function of the relative abundances of each species (546 species in TEDDY, 597 in DIABIMMUNE) at each sample collection. To evaluate the model performance, we examined the correlation between predicted and true ages and found that both models performed quite well (Spearman: 0.91 and 0.83; Fig. S4A and B), suggesting that there is a strong relationship between age and the developing microbiome beyond an age of 3 years. Model performance remained high, even when incorporating only the top 11 or 10 most important species (Spearman: 0.86 and 0.78; Fig. S4C and D, respectively). We wanted to assess whether each model contained a similar set of important species despite being trained on completely different populations. To that end, we identified the importance of age-discriminatory species for each non-CF data set from the random forest feature importance metric and assessed correlations between the two lists. There is a high correlation between the importance scores across all species. Furthermore, 29 of the 50 most important species are shared between the two models (Spearman’s rho = 0.75; Fig. 2A and B). A notorious problem with statistical models is that of extrapolation; often, models perform very poorly when tested on a different population because of underlying differences in data structure between the two populations. Despite this challenge, we found that each model performed well when tested on the other population (Spearman: 0.81 and 0.75; Fig. S4E and F), suggesting that a similar set of species defines microbiota age progression in different populations. In summary, we identified a set of age-discriminatory bacterial species whose prevalence and relative abundance are sufficient for the accurate cross-study prediction of non-CF infant age, essentially serving as biomarkers. To our knowledge, this is the first report of a cross-study validation of a microbiota age model, and our results demonstrate that such models can be highly robust and reproducible across non-CF data sets.

Next, we evaluated whether age-model species were detectable in the Dartmouth CF infant samples. Several top age-model species exhibit similar prevalence between non-CF infants and infants with CF, including *Blautia wexlerae* and *Eubacterium hallii*. However, other age-model species, including the species with the highest interstudy model importance, *Faecalibacterium prausnitzii*, exhibited an increase in prevalence in non-CF infants to nearly 100% by 24 months of age, yet remained at low prevalence in infants with CF (Fig. 2C; Table S5). To assess if differences in the prevalence and relative abundance of top age-model species resulted in altered relative microbiota age in infants with CF, we applied non-CF infant age models to our Dartmouth CF samples in order to calculate the relative microbiota age for each sample following previous methods (7, 26). Remarkably, relative microbiota age for CF exhibited negative values that strongly negatively correlated with true age to 18 months, regardless of the model used (Fig. 2D and E; TEDDY age model *P* = 7.56 × 10^−19^, DIABIMMUNE *P* = 3.69 × 10^−20^). After 18 months, CF relative microbiota age remained negative but did not further decrease. These findings were consistent with age models generated for both non-CF data sets with only the top 11 or 10 age-discriminatory taxa, respectively (Fig. S4G and H; TEDDY age model *P* = 6.14 × 10^−18^, DIABIMMUNE *P* = 2.81 × 10^−16^), suggesting that the prevalence and relative abundance of a limited number of key taxa are biomarkers for age and can differentiate between health and disease. Together, these data indicate that the CF infant gut microbiome exhibits a persistent developmental delay in maturation that is exacerbated over time up to 18 months of age and does not recover by 36 months.

### Cumulative antibiotic exposure is negatively associated with microbiome maturation in infants with CF

Our relative microbiota age model analysis revealed a delay in maturation of the CF infant gut microbiome compared to non-CF infants associated with a defect in the prevalence and relative abundance of key age model species, such as *F. prauznitzii*. We considered possibilities that might explain this delay in maturation. First, the gut microbiome of infants with CF may simply be compositionally distinct from that of non-CF infants at later timepoints, with a paucity of species typically important for maturation. In support of this concept, dietary differences, disease exacerbations, and therapeutic exposures might together contribute to shift the microbiome away from a stereotypical developmental trajectory through enrichment for species, such as *E. coli (*25–27, 40, 41). An alternate and non-mutually exclusive hypothesis partially supported by our analysis of beta-diversity (Fig. 1D and E) is that the CF gut microbiome might initially resemble that of non-CF infants in early life but remain entrenched in an immature state at later timepoints when gut microbiome developmental maturation has continued in non-CF counterparts.

**Fig 3:**
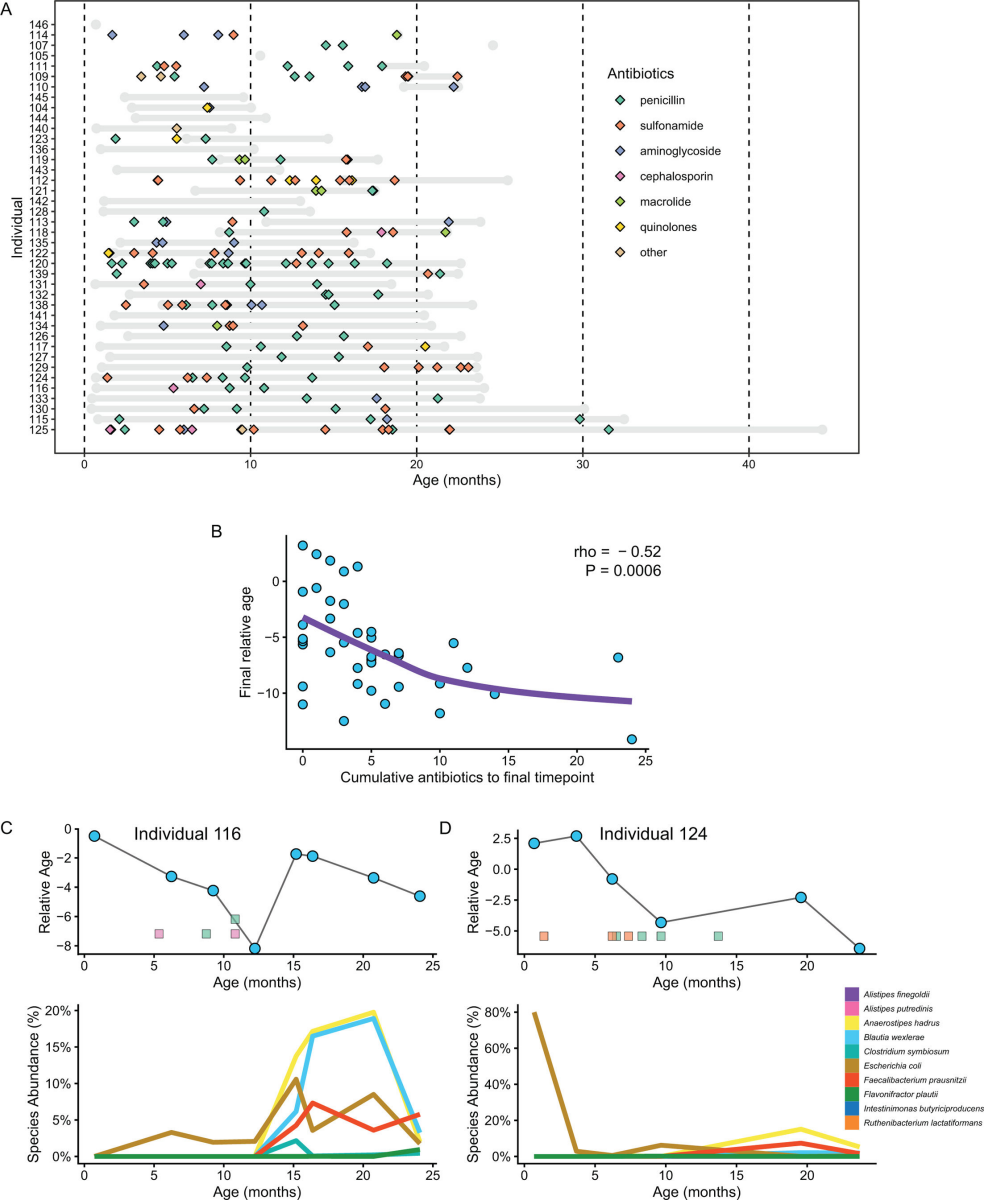
Persistent decline in microbiota relative age in infants with CF infants. (**A**) Schematic depicts all individuals in the Dartmouth CF infant cohort. Gray bars indicate the time period covered by sequencing of fecal samples, and colored diamonds represent the timing of each exposure by each indicated antibiotic class in key. (**B**) Relative microbiota age at the final sequencing timepoint for individuals in the Dartmouth cohort using a TEDDY-based model plotted against cumulative antibiotic exposures. (**C and D**) Relative microbiota age progression and age model species relative abundance from representative individuals from the Dartmouth CF cohort. Antibiotic exposures are indicated as for panel **A**.

To explore the first possibility, we decided to focus on antibiotic exposure in the Dartmouth CF cohort. Antibiotics impact the gut microbiome of infants either through effects on stability or composition, though the magnitude and consequence of the effect appear to differ across studies (3, 37, 42, 43). We found that the degree of antibiotic exposure in the Dartmouth CF cohort varied widely, with a few unexposed individuals and many with substantial exposure (mean number of exposures: 7.6, mean age of first exposure: 6.8 months; Fig. 3A; Table S6). Examination of the relationship between cumulative antibiotic exposure and microbiota maturation (using final microbiota relative age score) revealed a remarkably strong negative correlation across individuals (rho = −0.52, *P* = 0.0006). More granular analyses revealed individualized responses. For example, in some cases, such as individuals 116, 124, 130, and 135 (Fig. 3C and D; Fig. S5), early antibiotic exposure corresponded with a decline in relative microbiota age. However, in these cases, cessation of antibiotic exposure preceded a rebound in age model species relative abundance and an increase in relative microbiota age. Conversely, other individuals exhibited initially high relative microbiota age scores corresponding with detectable levels of some age model species (notably *B. wexlerae*, *F. prausnitzii*, and *R. lactatiformans* for individual 129 and *B. wexlerae* and *A. hadrus* for individual 139) until the first exposure to antibiotics. It is important to note that some individuals were not exposed to antibiotics yet exhibited declines in relative microbiota age. Together, these findings implicate cumulative antibiotic exposures contribute to microbiota maturation defects in CF.

### Unsupervised clustering reveals that microbiota of infants with CF fails to transit through distinct community types

**Fig 4:**
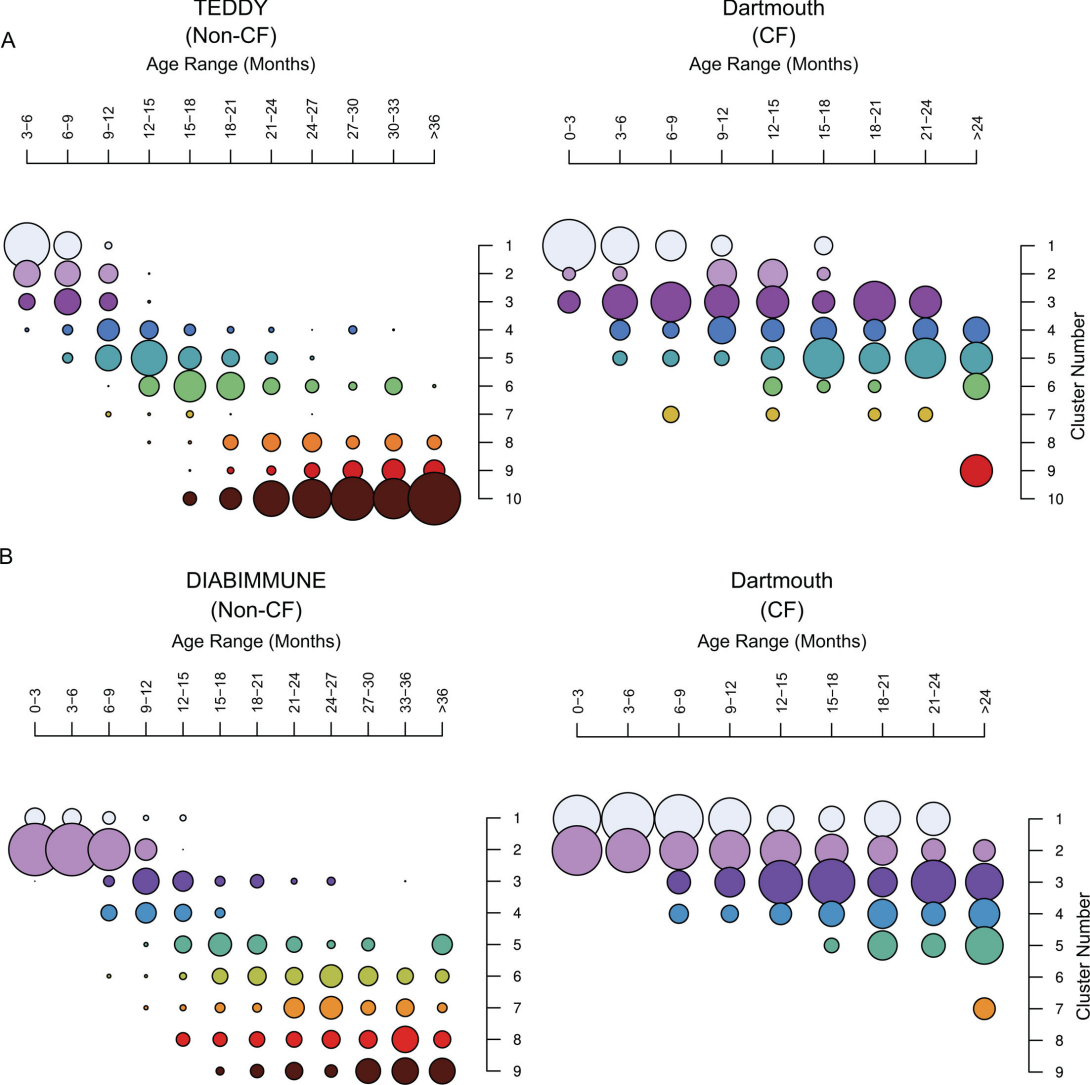
DMM clustering shows delayed development of the CF microbiome compared to non-CF controls. (**A**) Dirichlet multinomial mixture (DMM) clustering of infants from the TEDDY (left) and Dartmouth (right) cohorts. A DMM model was trained on data from TEDDY and applied to cluster data from Dartmouth. Samples were binned into 3-month age bins, and clusters were ordered based on the age bin where they are most abundant. Each cluster is denoted by a separate color (*n* = 1,429). (**B**) DMMs of infants from the DIABIMMUNE (left) and Dartmouth (right) cohorts plotted in the same way as in panel A (*n* = 1,335). Circle size depicts the number of samples in each cluster at each age bin.

To understand in greater detail the nature of the maturation defect in CF, we employed Dirichlet multinomial mixtures (DMM) probabilistic modeling to cluster non-CF microbiomes by community types based upon the relative abundance profiles of a limited set of species identified as most important (99% of model performance, 25 species for TEDDY and 21 species for DIABIMMUNE) for both microbiota age models trained on each data set (Fig. 2A). We identified 10 discrete clusters for the TEDDY data set and nine clusters for DIABIMMUNE (Fig. S6). As previously reported, we found that DMM clusters generated from non-CF infant metagenomes fell into discrete phases corresponding to stereotypical progression through ontogeny, with a “developmental” phase between 0 and 12 months, a “transitional” phase between 12 and 18 months, and a “stable” phase between 18 and 36, respectively (Fig. 4A and B; Fig. S6A, B, S7A and B; Tables S7 to S10). Next, we assigned CF microbiome samples to clusters by using the highest probability from the non-CF model. During the developmental phase (0–12 months), we find a small skew between CF and non-CF samples in occupying the developmental clusters (67% CF vs 82% non-CF with the TEDDY model, 66% CF vs 42% non-CF with the DIABIMMUNE model). Remarkably, we found that this difference in cluster occupancy persists in CF past the transitional phase. Microbiomes from infants with CF fail to progress past clusters that are representative of the non-CF transitional phase (20% of non-CF samples in the stable phase remains in clusters dominant in the transitional phase compared to 61% of CF samples in the TEDDY model, 9% non-CF vs 47% CF in the DIABIMMUNE model). Since the CF cohort possessed fewer samples at later timepoints, we performed additional analysis on age-matched samples and found similar patterns (Fig. S8A and B). To investigate if this pattern of microbiome developmental progression was unique to CF or was also observed in a separate pediatric disease, we used the same species-level taxonomic profiling and cluster assignment method to examine metagenomic samples collected from a cohort of infants prior to the onset of celiac disease (CD) (44). Unlike CF samples, those from infants with CD could be assigned to all non-CF clusters and did not display an age-dependent stall in cluster progression like that observed for CF (Fig. S9A and B). We conclude that our DMM cluster assignment method identifies the persistence of a transitional microbiome state during infant development in CF.

### Phase-level comparisons reveal a low-density microbiota signature in CF

**Fig 5:**
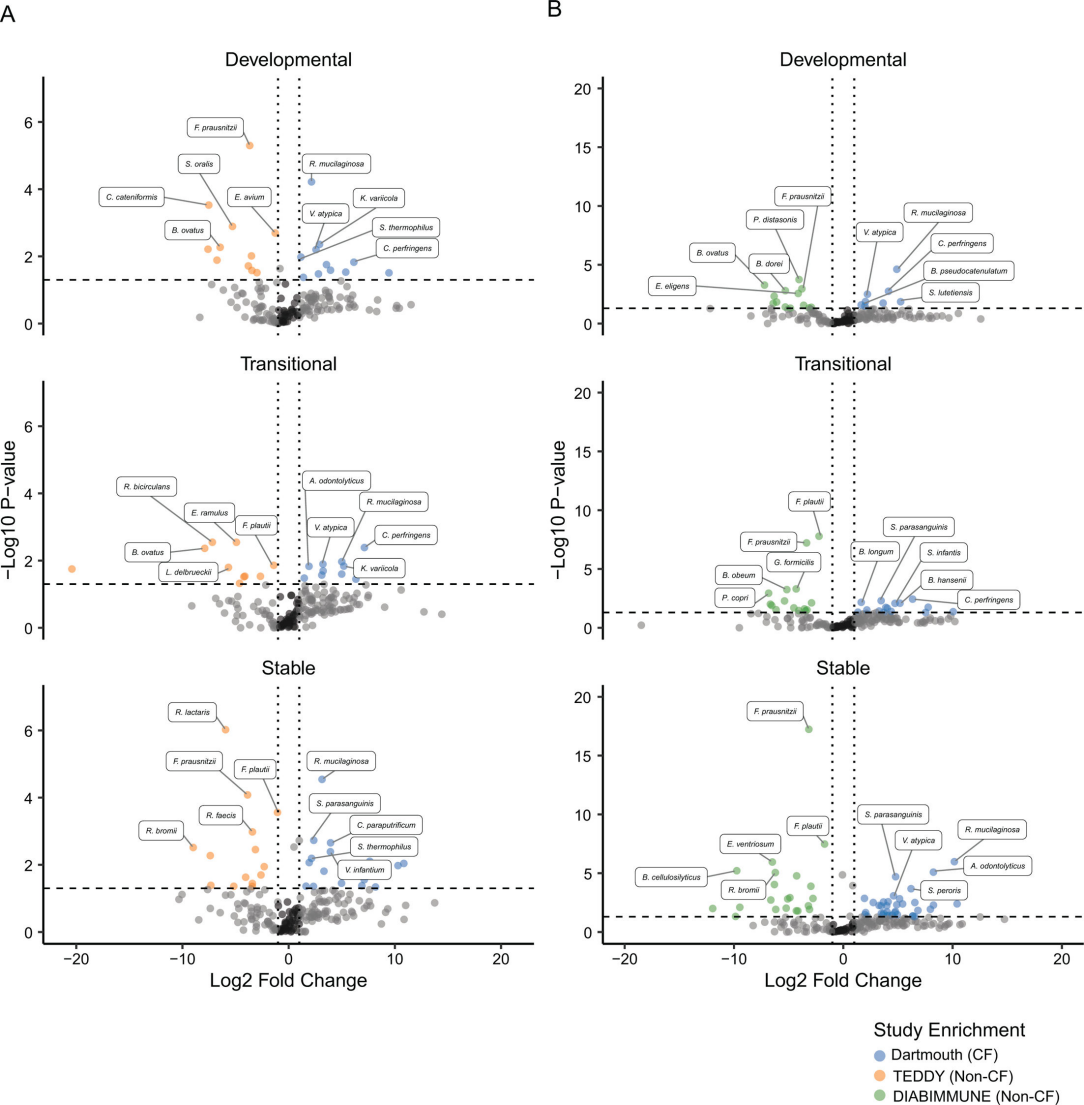
Phase-level differences in species relative abundance between CF and non-CF infants. (**A**) Volcano plots of species enrichment in Dartmouth cohort compared to TEDDY cohort at each of the three developmental phases (developmental = 0–12 months, transitional = 12–18 months, stable = 18–36 months). Species whose relative abundance differs significantly are colored based on which study they are enriched in (Dartmouth = blue, TEDDY = orange, *P* < 0.05, linear mixed-effects modeling, and log2FC < −1 or > 1). The top five most significant species (based on *P* value) for each study and each phase are labeled (*n* = 1,436). (**B**) Volcano plots of species importance in Dartmouth cohort compared to DIABIMMUNE cohort at each of the three developmental phases (developmental = 0–12 months, transitional = 12–18 months, stable = 18–36 months). Species of statistical significance (*P* < 0.05, linear mixed-effects modeling, and log2FC < −1 or > 1) are colored based on which study they are enriched in (Dartmouth = blue, DIABIMMUNE = green). The top five most significant species (based on *P* value) for each study and each phase are labeled (*n* = 1,344).

**Fig 6:**
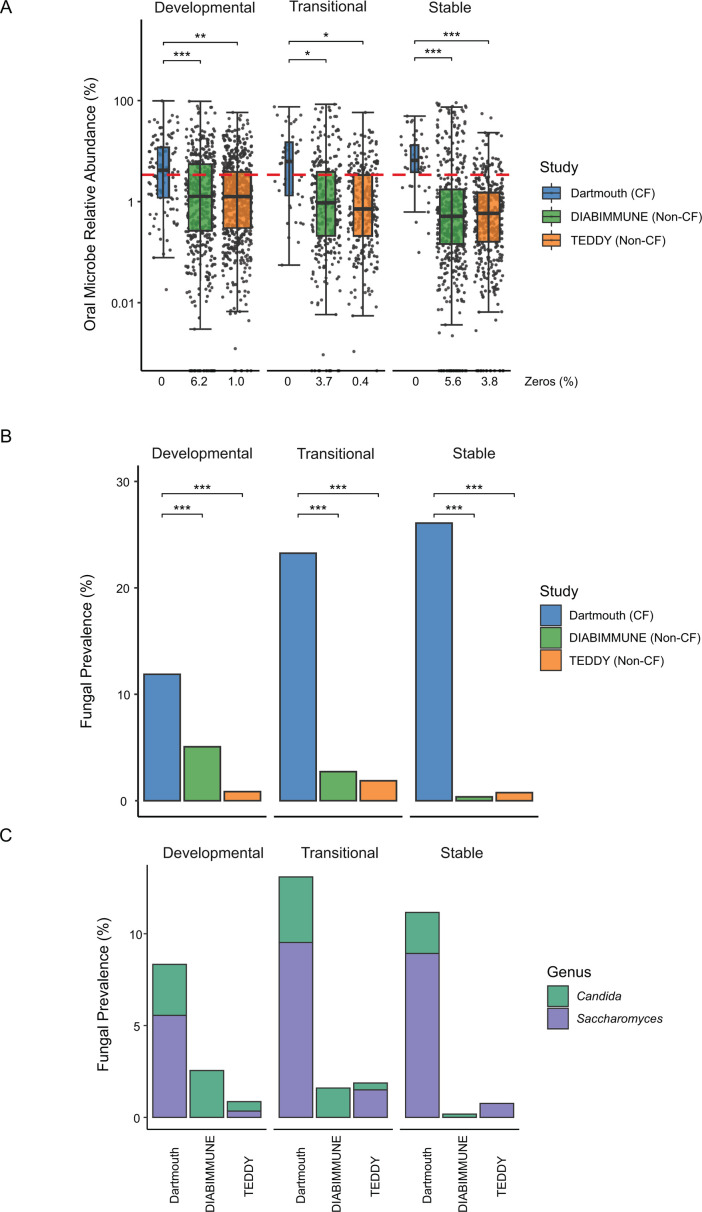
Oral and fungal relative abundance is higher in CF infants compared to non-CF infants in early life. (**A**) Relative abundance of oral microbes in Dartmouth (blue), DIABIMMUNE (green), and TEDDY (orange) cohorts at each developmental phase (developmental = 0–12 months, transitional = 12–18 months, stable = 18–36 months). Red line signifies 3.4% relative abundance. *P < 0.05, **P < 0.01, and ***P < 0.001; linear mixed-effects modeling, *n* = 2,590. (**B**) Fungal prevalence in the Dartmouth (blue), DIABIMMUNE (green), and TEDDY (orange) cohorts at each developmental phase (developmental = 0–12 months, transitional = 12–18 months, stable = 18–36 months) *P < 0.05, **P < 0.01, and ***P < 0.001; linear mixed-effects modeling. Abundance was first collapsed by genus, and prevalence was calculated per individual. (**C**) Fungal prevalence by genus in the Dartmouth, DIABIMMUNE, and TEDDY cohorts at each developmental phase (developmental = 0–12 months, transitional = 12–18 months, stable = 18–36 months).

We next assessed microbiome compositional differences by identifying species most differentially abundant in CF at each phase of development through separate comparisons to the TEDDY and DIABIMMUNE data sets (Tables S11 and S12). Species significantly less abundant in infants with CF and more abundant in non-CF infants at each phase included important age-model species notable producers of host-beneficial metabolites, such as *F. prausnitzii* and *Ruminococcus bromii* (Fig. 5A and B) (45, 46). Additional species identified to be significantly depleted from the CF gut microbiome include prominent members of the genus *Bacteroides*, consistent with previous reports that these species are notably less abundant in CF (22, 26). Species identified to be more abundant in CF across phases and in comparison to non-CF data sets included potential pathogens like *Clostridium perfringens*, as well as many species associated with the oral and upper respiratory microbiome. An enrichment of oral species in the fecal microbiome has been previously reported to be associated with inflammatory bowel disease, as well as low-density gut microbiota caused by antibiotic exposure (47). In line with this, we find that the total relative abundance of oral species is significantly higher in CF than for non-CF infants at each phase of microbiome development, with a majority of samples exceeding an abundance threshold of 3.4% found to be associated with low-density microbiota states (Fig. 6A; Table S14) (47).

An increase in fungi in the gut has also been reported for microbiomes with low bacterial density (47). We assessed fungal prevalence in our data sets and found elevated fungi in CF compared to either non-CF data set, with frequent detection of *Saccharomyces* and *Candida*, both of which have independently been associated with inflammatory bowel conditions (Fig. 6B and C; Tables S15 and S16) (48, 49). Together, these findings implicate a low-density gut microbiota in CF with corresponding loss of beneficial species that include prominent contributors to non-CF microbiota relative age.

### Altered microbiota functional capacity in CF

Differences in the composition of the microbiota can result in altered functional potential between groups. To assess if this is the case, we next evaluated whether alterations in the fecal microbiota of infants with CF resulted in significant differences in metabolic potential and functional pathway abundances through comparisons to both TEDDY and DIABIMMUNE (Fig. S10 and S11; Tables S17 and S18). We used hierarchical clustering of the top most-significant functional modules from comparisons of CF to each non-CF data set separately. This analysis clearly showed separation by health status instead of sample age, with the exception of the CF stable phase, which clustered together with the TEDDY transitional and stable phase samples. Pathways determined to be the most significantly enriched in CF at all phases include fatty acid biosynthesis and the dicarboxylate–hydroxybutyrate cycle and reductive acetyl-CoA (Wood–Ljundahl) pathways, indicating a dramatic shift in metabolic capacity in CF previously associated in non-CF infants with the pre-weaning state (2, 50, 51). In contrast, modules involved in the adult microbiome-associated vitamin B7 biotin biosynthesis pathway were significantly depleted in CF samples across phases (51). We also assessed the functional contribution of taxa statistically determined to be more abundant in either non-CF or CF infants for each phase of microbiome development. Notably, species more abundant in non-CF infants were major contributors to functional pathway abundance (Fig. S12A and B), mirroring the high relative abundances of these species (Fig. S12C and D). *F. prausnitzii* was particularly notable as a top functional pathway contributor across phases and in both non-CF data set comparisons. In contrast, species significantly more abundant in CF exhibited minimal contributions to functional pathway abundance and were on average low abundance (Fig. S11A through D). Together, these findings demonstrate that the alterations in taxonomic composition we observe between non-CF infants and infants with CF reflect dramatic differences in functional pathway abundance.

### Reduced strain-level diversity of *Faecalibacterium prausnitzii* in CF

**Fig 7:**
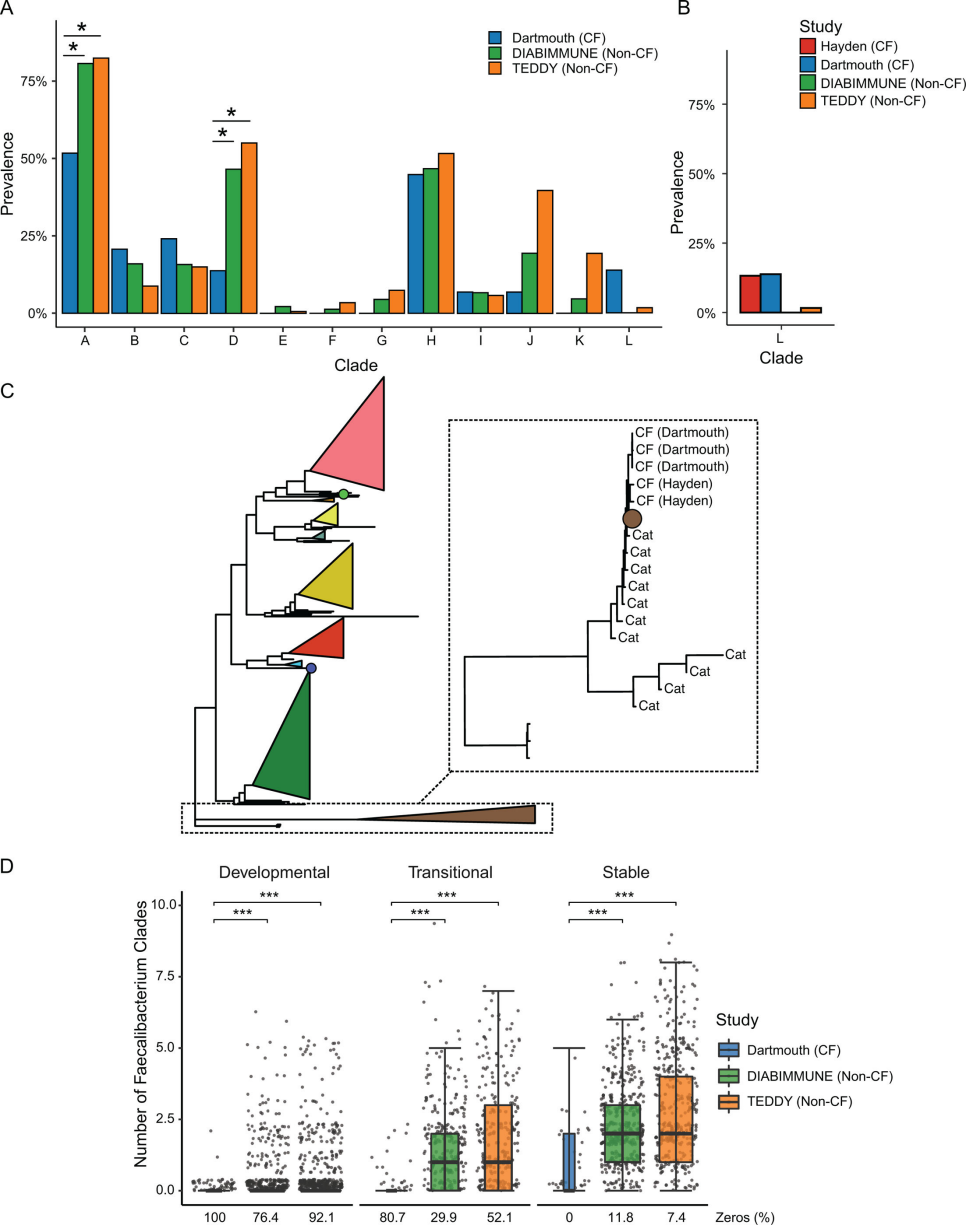
*Faecalibacterium prausnitzii* strain diversity is altered in CF infants compared to non-CF controls. (**A**) Overall prevalence of *F. prausnitzii* clades in CF and non-CF cohorts (*FDR ≤ 0.05, linear mixed-effects modeling). (**B**) Clade L prevalence across CF and non-CF cohorts. (**C**) Phylogenetic tree of *F. prausnitzii* strains. The tree contains data derived from publicly available reference genomes, as well as sequences reconstructed from feline metagenomes and human CF infant metagenomes (Dartmouth and Hayden). Detected strains were assigned to clades based on the presence of clade specific marker genes and collapsed into triangle form. Black box highlights strains in clade L and is magnified in the inset box to the right depicting strains inferred from feline metagenomes. (**C**) Number of *F. prausnitzii* clades identified in Dartmouth (blue), DIABIMMUNE (green), and TEDDY (orange) cohorts at each developmental phase (developmental = 0–12 months, transitional = 12–18 months, stable = 18–36 months). ***P < 0.001, linear mixed-effects modeling (*n* = 2,590).

We chose to investigate the CF-depleted butyrate producer *Faecalibacterium prausnitzii* in greater detail at the strain level due to its importance in our microbiota age-model analysis (Fig. 2), its significant contribution to functional pathway abundance in non-CF data sets (Fig. S12), and its well-established role in gut health and development (45). *F. prausnitzii* has previously been identified as a biomarker of intestinal health and may limit inflammation in part through production of the SCFA butyrate (52). Healthy infants are typically colonized early in life with *F. prausnitzii*, with the relative abundance and strain diversity increasing over age (53). We, therefore, assessed *F. prausnitzii* diversity in infants with CF compared to the TEDDY and DIABIMMUNE non-CF cohorts. We detected all human-associated *F. prausnitzii* clades in at least one infant metagenome, with the prevalence of clades B, C, D, G, and I increasing with age (Fig. S13A and B). Comparing *F. prausnitzii* diversity across samples, we found all clades to be more prevalent in the non-CF cohorts, except for clade L, which is more prevalent in both distinct CF cohorts (Fig. 7A and B). Interestingly, clade L is predominantly associated with pets, including canines and felines (53, 54), and pet-associated *F. prausnitzii* has been reported to colonize humans (55, 56). Phylogenetic analysis of *F. prausnitzii* clade L strains, including those detected in CF infant samples, reveals relatedness between human- and pet-derived strains (Fig. 7C). These data suggest the CF infant gut is more permissive to colonization by non-human strains of *F. prausnitzii* than that of non-CF infants. Finally, since the decreased within-sample clade diversity of *F. prausnitzii* is associated with disease states, including inflammatory bowel disease (53), we compared *F. prausnitzii* diversity between non-CF and CF samples for each phase. We detect significantly more clades in non-CF samples than in CF for all phases (Fig. 7D). Together, these results demonstrate that a prominent feature of the CF infant microbiome is the dramatically altered prevalence and diversity of *F. prausnitzii*.

## DISCUSSION

Our findings demonstrate that the gut microbiota of infants with CF fails to undergo typical developmental maturation, instead remaining entrenched in a transitional-like community state. This conclusion is based upon our age-model analysis comparing CF infant microbiome development to two of the largest published publicly available non-CF infant metagenomic data sets, TEDDY and DIABIMMUNE. Cross-validation of age-model species importance demonstrates that key taxa are reproducibly associated with chronological age across non-CF data sets that are compositionally distinct with major differences in prominent phyla, such as Bacteroidota and Actinomycetota. While the prevalence and relative abundance of species like *F. prausnitzii* therefore represent a biomarker of age, it is important to note that many important age-model species are themselves found at high levels in the adult microbiome and have been implicated in vital functions, such as the production of SCFA and other metabolites, that impact host physiology at both local and distal sites (57, 58). Altered microbiome maturation, therefore, is likely to reflect fundamental consequences for both taxonomic composition and function.

We identified an enrichment of oral-derived bacterial taxa at all developmental phases in infants with CF. A similar signature has been implicated in a low-density gut microbiota state that is associated with antibiotic usage (47). Our findings of a negative association between cumulative antibiotic exposure and relative microbiota age suggest that antibiotics may be causal in driving dysbiosis. However, several observations imply that the relationship between antibiotic exposure, *CFTR* deficiency, and microbiota maturation is complex. First, it is notable that mouse models of CF exhibit altered microbiota composition in the absence of antibiotic exposure or other external insults (32, 59). We also observed individuals in the Dartmouth CF cohort that lacked any antibiotic exposures yet exhibited severe microbiota maturation defects. Antibiotic exposure (particularly for treatment of lung infections) may itself be correlated with disease severity and microbiota maturation in CF correlates with delays in growth in the first year of life (26). Thus, while our data implicate antibiotics in driving at least some aspects of altered microbiota maturation, further investigation of prospective human cohorts or the use of animal models of CF will be needed to understand the causal underpinnings of CF-related microbiota alterations and their consequences for the host more fully.

In mice, experimental manipulation of gut microbiota density results in changes to host metabolism and immunological dysregulation (60). One potential consequence of reduced gut microbiota density is lowered concentrations of microbiota dependent metabolites that mediate beneficial effects for the host. Altered metabolite concentrations have been observed in infants with CF; however, whether or not these changes are due to alterations in taxonomic composition, altered density, or both remains an open question (61). Another consequence of low gut microbiota density is an increase in the abundance of fungi, and we observe a higher prevalence of fungal taxa, such as *Candida*, in infants with CF, consistent with evidence that people with CF are more likely than non-CF counterparts to possess anti-*Saccharomyces cerevisiae* antibodies (ASCA), which recognize oligomannose in the yeast cell wall, including that of *Candida* species (62). Intestinal colonization by *Candida* species is linked to inflammatory bowel diseases and the induction of Th17 responses both in the gut and in the lung; however, the impact of infant carriage of Candida in CF is unexplored (63). Future work will address the potential relationships between antibiotic treatment type and frequency, higher fecal relative abundance of fungi and oral bacteria, and microbiota maturation defects in CF.

The link between atypical microbiome maturation and intestinal inflammatory diseases in infants remains generally underexplored. Our analysis of microbiome development in infants prior to onset of celiac disease revealed no obvious differences compared to non-CF development. While it is possible that impaired microbiome development is a unique feature of CF, an alternate possibility could be this signature reflects active disease during this time period. In CF, infants exhibit levels of fecal calprotectin that increase over time and other markers of inflammation from very early in life, and it is probable that active disease in CF initiates at or even before birth (64). Although CD and other inflammatory intestinal diseases typically manifest later in childhood at the earliest, it is possible that disease occurring during the developmental or transitional phase of development (rather than after the attainment of stable phase) might be associated with patterns maturation delay similar to what we observe in CF. It is notable that the infant disease kwashiorkor (etiologically unrelated to CF and caused by severe malnutrition) is also linked to maturation delays in microbiome development and coincident intestinal inflammation (65).

The gut microbiota are critical for early-life training of the developing immune system, and disruptions to the microbiota during this time period can lead to subsequent metabolic and immunologic dysregulation (10, 66). In mice, microbiome dynamics during the weaning period are necessary for a transient inflammatory response known as the weaning reaction. Suppression of microbiome dynamics (specifically the bloom in gram-positive SCFA producers during and following weaning) prevents the weaning reaction and predisposes mice to inflammation and pathogen susceptibility via restriction of peripheral regulatory T cell expansion (10, 13). We find that infants with CF harbor populations of Gram-positive SCFA producers like *F. prausnitzii*, which fail to expand in relative abundance over the first 3 years of infancy and consequently do not diversify at the strain level as in non-CF infants. The potential for a link between impaired microbiome development in CF and deleterious immunological sequelae warrants further investigation. Future mechanistic work investigating the link between microbiota age delay and disease in CF will benefit from the experimental framework leveraging gnotobiotic models and dietary interventions laid out by other fields (67). For example, nutritional supplementation in CF targeted at promoting the growth of keystone age-model species like *F. prausnitzii* may be a promising avenue to overcome microbiome age delay in hopes of improving health.

Our study has some limitations, which we describe here in part to motivate future studies. First, while we gained significant insight, generalizability, and power in our use of publicly available large sequencing data sets, we lacked a non-CF control cohort recruited at our Dartmouth hospital. Such a cohort would have provided additional important context with which to compare our other data sets. Second, due to the retrospective nature of our study, in some cases, we only had access to genomic DNA and not the physical stool samples. This prevented us from empirically determining the absolute abundance of the microbiota, which would have greatly enhanced our ability to interpret our findings of increased oral bacterial abundance and the association of antibiotic exposure with delay in microbiota maturation. Finally, due to our lack of access to physical stool samples, we were unable to perform metabolomic analyses that could have provided insight into the functional consequences of gut microbiota dysbiosis in infants with CF.

### Conclusions

In summary, we identify key features of the developing gut microbiota that differ between infants with CF and non-CF counterparts, including the prevalence, relative abundance, and strain-level diversity of species that are diagnostic biomarkers of microbiota age. The consequence of these differences is that the CF infant gut microbiota experiences a persistent delay in maturation that is exacerbated past the first year of life and fails to recover. This is accompanied by elevated levels of oral-derived species reminiscent of other intestinal inflammatory diseases. Future work should investigate the causes and consequences of microbiota age delay in CF with an ultimate goal of identifying microbiota-directed therapeutics.

## MATERIALS AND METHODS

### New CF cohort sample collection

This study was approved by the Dartmouth College Committee for the Protection of Human Subjects (CPHS study #00021761). Fecal samples were collected longitudinally from 40 infants with CF from 13 days up to 45 months of age during a time period spanning the years 2009–2019. A total of 190 samples were collected and analyzed. Each subject had between 1 and 11 samples collected, with a median of five samples collected per subject. Fecal samples were collected by parents and initially stored in a home freezer prior to transfer to clinics throughout New England during routine visits with clinicians. At the clinic, the samples were stored at −80°C until they were transported to the lab to be aliquoted, stored in −80°C, and later processed for sequencing.

### Non-CF control cohort selection

Longitudinal control studies were selected based on the sample size and age range of individuals. We specifically selected cohorts ranging between 0 and 3+ years of life and who had large sample sizes of infants that had at least two samples collected over that time frame. Hayden et al.’s work was selected to compare CF cohorts, as well as our CF cohort to an age-matched non-CF cohort within the first year of life (26). All samples from the DIABIMMUNE study (3, 36, 38) and 10% of the individuals followed in the TEDDY study (6, 37) were selected as large non-CF cohorts extending into the third year of life as an extended age matched control to our cohort. To obtain a representative number of samples from the TEDDY study, 88 individuals were randomly selected and evenly distributed from Europe and the United States to account for any possible location bias. All collected samples from each individual were used in subsequent computational analysis.

### DNA isolation and sequencing

Infant fecal samples stored at −80°C were thawed and aliquoted to ~100 mg in Eppendorf tubes prior to DNA extraction. DNA was isolated using the Zymo Quick-DNA Fecal/Soil Microbe Miniprep Kit (Cat #D6010). DNA concentrations were measured via a Qubit fluorometer, and sequencing was performed on an Illumina NextSeq platform (Microbial Genome Sequencing Center, now SeqCenter). Sequencing produced an average of 20,320,837 reads, and 97.8% of samples had at least 12 million reads.

### Taxonomic profiling

After sequencing, samples were filtered and trimmed when applicable using BBDuk, trimming bases with a quality score below 12 and filtering out reads below 20 bases. BBMap was used to remove human DNA from each sample prior to taxonomic profiling (68). Quantification and profiling of taxa in each sample were performed using MetaPhlAn3 (69). Briefly, MetaPhlAn3 identifies members of the microbiota in each sample using a database comprising unique marker genes specific to each individual species, which are used to quantify the abundance of each microbe detected in sequencing (70). We used default settings and a minimum read length of 30. For the phylum-level plots in Fig. 1, samples were collapsed and summed by phylum classification. After filtering for top phyla, samples were normalized to 100% and rank-ordered by the abundance of Proteobacteria, with *E. coli* abundances determined for each sample prior to plotting. Raw taxonomic relative abundances were used for all subsequent analyses. The DMM clustering method (see below) required counts, and we estimated the number of counts mapping to each lineage with the rel_ab_w_read_stats flag.

### Statistical analysis

Statistics were performed using R versions 3.6.3 and 4.1.2 (R Core Team 2021). Significance was considered at *P* < 0.05, unless otherwise indicated. Multivariate analysis was performed using PERMANOVA tests implemented by adonis in vegan version 2.5.7 (71). Multidimensional scaling (principal coordinate analysis) of taxonomic profiles was completed using unweighted Unifrac in phyloseq version 1.38.0, and the top three species were identified by calculating the mean Euclidean distance of the vectors for each species (72). Alpha diversity was calculated using Shannon diversity index using custom code. Hypothesis testing was performed using a one-sided Wilcoxon rank-sum test where indicated. Multiple hypothesis correction was performed using the Benjamini–Hochberg procedure where applicable. To identify significant associations between KEGG modules and developmental phases, we used MaAsLin2 for linear mixed-effects modeling using module and phase as fixed effects and a minimum abundance of 0.01%, and the significant-results file from the automated output was used for further analysis (73). To account for the fact that some of our observations were measurements from the same individuals over time and hence not strictly independent, we used mixed-effects models with “individual” as a random effect to evaluate significance. Specifically, for contingency tables of presence/absence, we used a generalized linear mixed-effects model with a binomial response as implemented in the glmer function of the lme4 package in R (74). For count data, we used a generalized linear mixed-effects model with a Poisson response. For abundance data that did not neatly follow a Gaussian distribution, we used a quantile linear mixed-effects model as implemented in the lqmm function of the lqmm package in R (75).

### Relative microbiota age analysis

We implemented the age model as has been described previously (7, 26). In brief, we used regularized random forests (RRF package in R) to predict the age of a microbiome sample from the relative abundance of microbiome species (calculated by MetaPhlAn3). Conceptually, the relative microbial age is the residual of the model prediction, but there is an additional step to correct for the significant saturating effect in the predicted model age. Specifically, we used a third-degree spline to model the relationship between the true and predicted ages, then we took the difference between the predicted age from the random forest and the prediction from the spline. The importance of each species was calculated using the random forest approach to variable importance (the decrease in residual sum of squares when splitting on the variable, averaged over all trees). We identified a subset of species that gave either 95 (11 or 10 species for TEDDY and DIABIMMUNE, respectively) or 99% (25 or 21 species for TEDDY and DIABIMMUNE, respectfully) of maximum performance by step-wise addition of species in order of decreasing importance until we reached the performance cutoff (either 95 or 99%). The performance of the model was evaluated using the Spearman correlation between the predicted and actual ages of the sample. All performances were evaluated in a 10-fold cross validation within each respective data set.

### Dirichlet multinomial mixture modeling

We followed previous methods to cluster developing infant gut microbiome data using the Dirichlet multinomial mixture (DMM) approach as implemented in the DirichletMultinomial package in R (6, 76). We provided the clustering algorithm with a subset of species to focus on the most important species for development. Specifically, we only considered the limited set of species that produced 99% performance in the age model (see above). Alternative models trained with either the top 25 most abundant species or all species with more than 0.01% average abundance gave qualitatively similar results (data not shown). DMM is a count-based method, and we used the count data estimated by MetaPhlAn3 (see above). We identified the optimal number of clusters using Bayesian information criterion; however, we found that the optimal cluster number was unstable with ±3 clusters changing over replicate runs. In each 3-month bin of data, we calculated the fraction of samples in each cluster. Bins with less than 15 samples were dropped due to statistical issues arising with low sample sizes. Clusters were ordered by the time point bin at which they were most abundant. We plotted the percentage of samples in each age bin as circles using the igraph package in R. Following previous methods, we dropped low abundance assignments (less than 4%) (6), but contrary to their work, we did not plot temporal transitions between clusters because we did not have sufficient sample density from each CF individual.

### Oral and fungal microbiota analysis

To calculate the oral bacterial fraction of metagenomic samples, we filtered taxa by the genera listed in Liao et al. and summed them on a per sample basis (47). Specific genera include the following: *Actinomyces, Leptotrichia, Camplyobacter, Fusobacterium, Neisseria, Corynebacterium, Rothia, Treponema, Veillonella, Prevotella, Streptococcus, Capnocytophage, Haemophilus*. Samples were then filtered for those containing greater than 3.4% oral species to find the percentage of total samples with a significant fraction of oral microbes (Table S11).

Fungal prevalence was calculated, and statistical tests comparing prevalence between non-CF and CF data sets were performed using linear mixed-effects modeling using the glmer function in the lme4 package in R.

### Functional pathway analysis

The functional capacity of each sample was analyzed using HUMAnN3, which aligns reads to a database of genes belonging to members of the microbiota (77). Briefly, identified genes are quantified at both the community and individual levels, which we then annotated using the Kyoto Encyclopedia of Genes and Genomes (KEGG). Community-level values were then collapsed into functional pathways and modules for further analysis. Statistical analysis was performed using MaAsLin2 using default parameters, with a minimum abundance of 0.01% (73). In short, MaAsLin2 uses linear modeling to account for covariates that may impact the desired result. Disease state and age were accounted for in this process and used as fixed effects. Heatmaps were generated using only modules noted in the significant results file output, and module abundances were rescaled to one to show contrasts on a per module basis.

To select the most significant species for the functional bar and box plots, significance testing was run at the species level using the taxonomic abundances generated by MetaPhlAn3 using the non-CF cohort as the control population. Significance was defined by having a *P* value < 0.05 and a log2FC of greater than one or less than −1. These values were then plotted in volcano plots to demonstrate species enrichment or depletion in the CF cohort. The top five most significant species in both the CF and non-CF data sets for each comparison and each developmental phase were labeled on the volcano plots and selected for further analysis. These species were filtered from the HUMAnN3 analysis, and these data were collapsed into KEGG pathways by finding the mean abundance among all samples where the functional abundance was >0, then summing the abundances for each module within each pathway for plotting. Prevalence was calculated by summing the total number of species with relative abundance > 0 divided by the total number of samples and multiplying by 100 to obtain percentage values.

### Strain-level analysis of *Faecalibacterium prausnitzii*

To detect *F. prausnitzii* strains, we used a set of clade-specific marker genes that were previously defined (53). We aligned metagenomic reads to the marker genes using bowtie2 (2.3.4.1, with parameters -a -N 1) and quantified the number of reads mapping to each maker, discarding multi mapping reads, reads that mapped at less than 95% identity, 50% of the read length, or below a quality score of 20. We determined the presence of a marker gene if it had at least 25 mapped reads, and we determined the presence of a strain if we could identify at least 10% of the marker genes. This is in contrast to previous methods that used the same cutoff of 10% of the marker genes to determine the presence of a strain but determined gene presence using a consensus sequence-based approach, specifically a consensus sequence with at least 95% identity over 50% of the gene length. We experimented with both approaches and found cases where divergent strains hovering around the 95% identity threshold would be missed by the consensus approach but successfully found by the count-based approach, and that these cases were convincing because of a large number of read counts. Additionally, the number of clades was quantified using the marker gene approach in each data set at each developmental phase. Statistical comparisons between the prevalence of clade were performed with the chi-squared test for the developmental stages or the Fisher’s exact test for the difference between CF and non-CF. Differences in the number of strains were assessed using a generalized linear mixed-effects model via the glmer function of the lme4 package in R.

We built a phylogenetic tree of *Faecalibacterium prausnitzii* by obtaining 367 reference genomes from GenBank in September 2022. We used prokka (1.13) to infer gene content and roary (3.13) to infer core genes (78, 79). For phylogenetic tree construction, we used core genes that were present in at least 50% of the genomes. To construct the tree, we identified marker genes in genomes using BLASTN against the prokka inferred gene sequences and filtered hits with less than 90% sequence identity and covering less than 90% of the length of the query and the hit. In addition, we included sequences inferred from CF-derived metagenome data sets, as well as previously sequenced cat metagenomes (54). *F. prausnitzii* marker genes in metagenomes were inferred by aligning reads to the marker sequences using bowtie 2, then constructing pileups using samtools (1.9) and creating a consensus sequence with the major allele at every position along the gene sequence (80, 81). Positions below a coverage of 5 or where the major allele was less than five counts higher than the minor allele were replaced with an N, as well as positions 10 bases from the start and from the end of the gene. We aligned all representatives of a marker gene individually using MAFFT (v7.505) and concatenated the individual alignments together. Using the concatenated alignment, we created a phylogenetic tree using RAxML using the GTRGAMMAI (general time reversible model with a gamma model of rate heterogeneity and with an estimate of the proportion of invariable sites) (82). Additionally, the number of clades was plotted in each data set at each developmental phase on a per sample basis. Statistics were performed using a generalized linear mixed-effects model via the glmer function of the lme4 package in R.

## Data Availability

The shotgun metagenomic samples generated in this study are publicly available through the Sequence Read Archive at the National Center for Biotechnology Information (NCBI) under BioProject accession number PRJNA955235. Other data sets analyzed in this study are listed in Table S3. The code used to analyze data and generate figures in this work is publicly available on GitHub at https://github.com/BenRossLab/Aging_CF.

## SUPPLEMENTAL MATERIAL

Figure S1 mbio.03420-24-s0001.pdf Relative abundances of select phyla differ between infants with CF and non-CF controls.

Figure S2 mbio.03420-24-s0002.pdf Gut microbiota from CF cohorts exhibit compositional similarity in the first year of life.

Figure S3 mbio.03420-24-s0003.pdf Altered microbiota compositional dynamics in CF infants.

Supplemental figures mbio.03420-24-s0004.pdf Figures S4 to S11.

Figure S12 mbio.03420-24-s0005.pdf Altered functional capacity in CF compared to non-CF cohorts.

Figure S13 mbio.03420-24-s0006.pdf *F. prausnitzii* increases in prevalence over time in non-CF infants.

Legends mbio.03420-24-s0007.docx Supplemental figure legends.

Supplemental tables, part 1 mbio.03420-24-s0008.xls Tables S1–S7.

Supplemental tables, part 2 mbio.03420-24-s0009.xls Tables S8–S18.

ASM does not own the copyrights to Supplemental Material that may be linked to, or accessed through, an article. The authors have granted ASM a non-exclusive, world-wide license to publish the Supplemental Material files. Please contact the corresponding author directly for reuse.
