# Supplementary figures and images for: Persistent delay in maturation of the developing gut microbiota in infants with cystic fibrosis
Source: mBio. 2025 Feb 13;16(3):e03420-24. doi: 10.1128/mbio.03420-24 (PMC11898760; doi:10.1128/mbio.03420-24)

Fig. S1

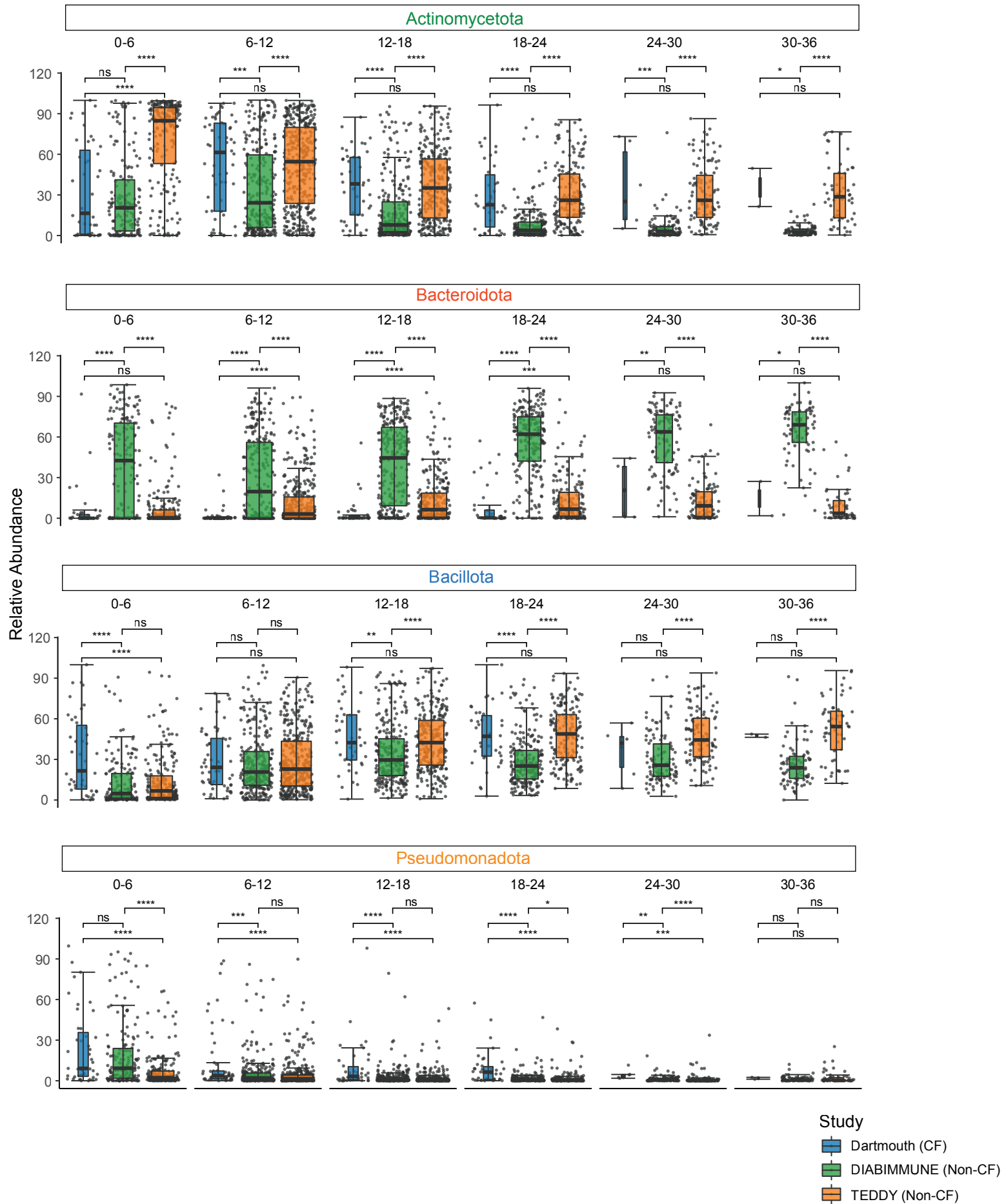

Supplement: Figure S1 — Relative abundances of select phyla differ between infants with CF and non-CF controls. [file mbio.03420-24-s0001.pdf]

Fig. S2

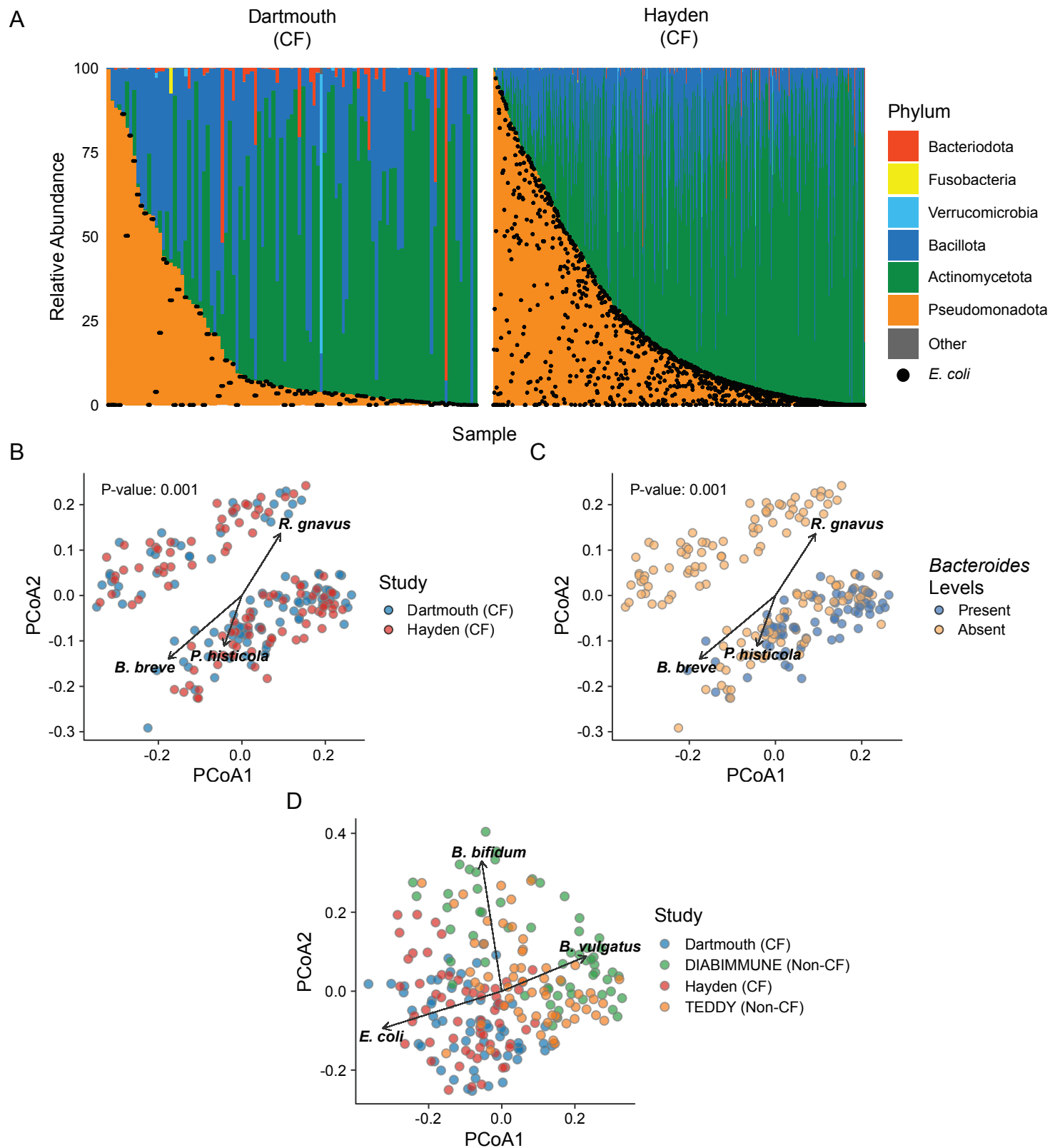

Supplement: Figure S2 — Gut microbiota from CF cohorts exhibit compositional similarity in the first year of life. [file mbio.03420-24-s0002.pdf]

Fig. S3

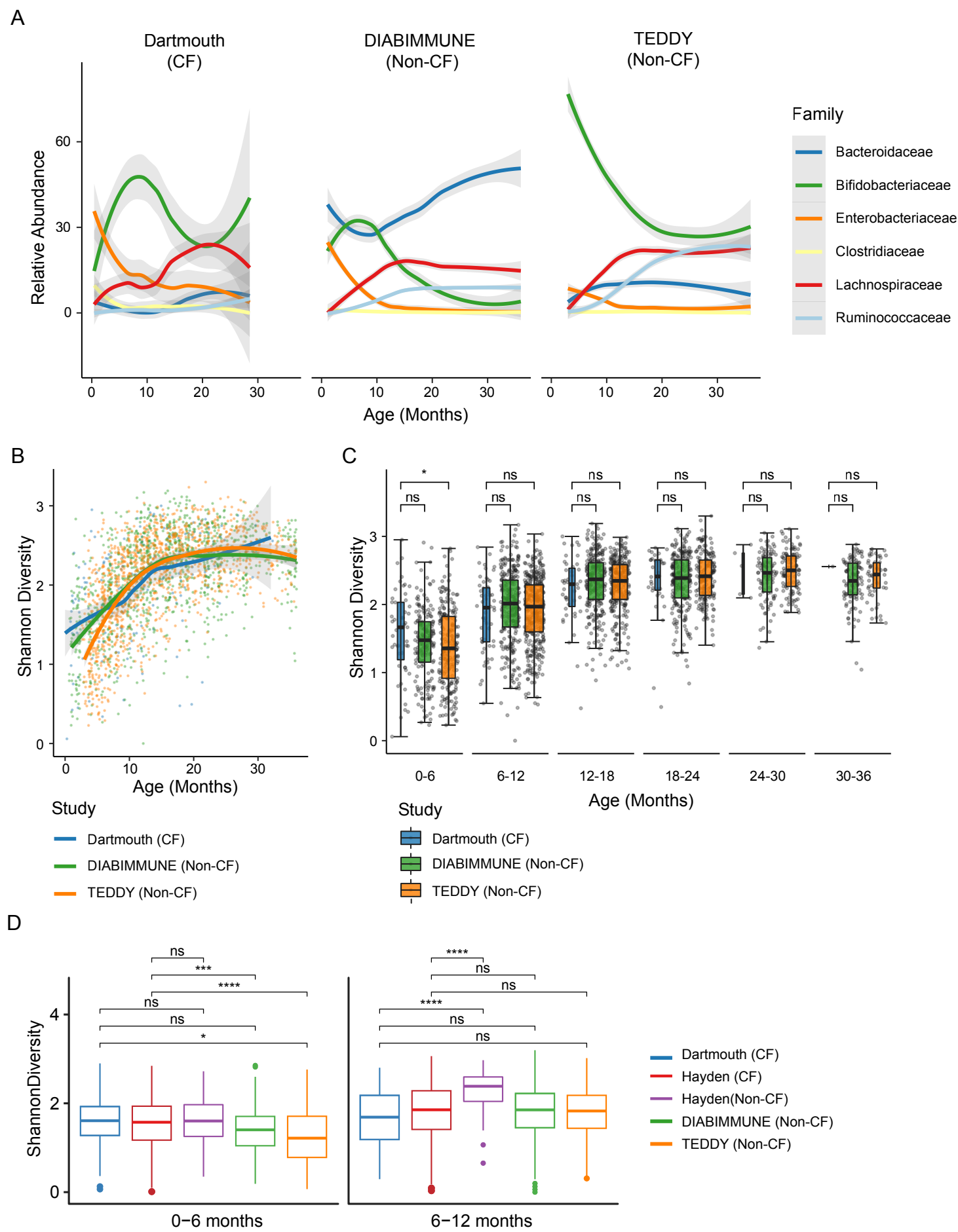

Supplement: Figure S3 — Altered microbiota compositional dynamics in CF infants. [file mbio.03420-24-s0003.pdf]

Fig. S4

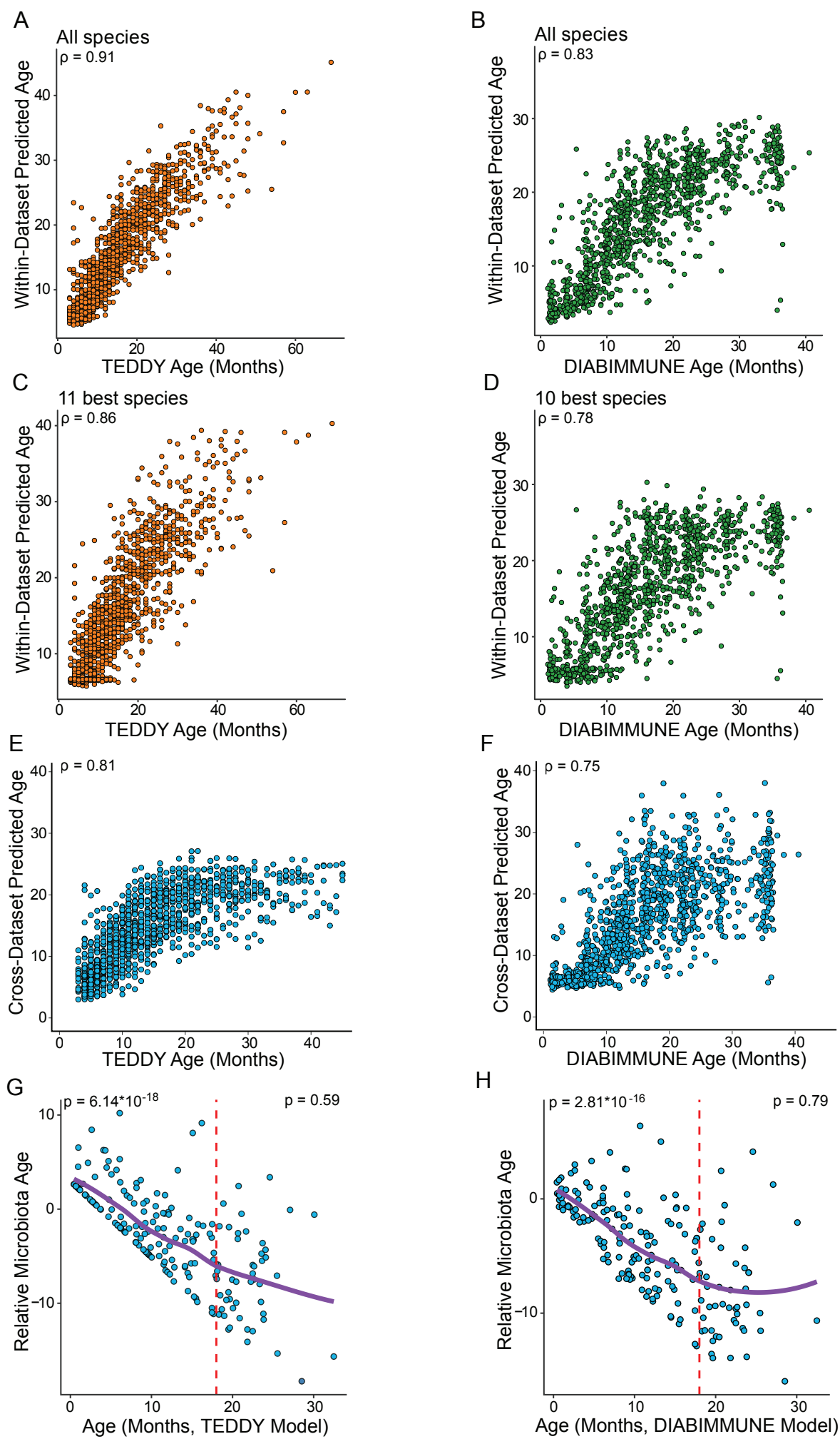

Fig. S5

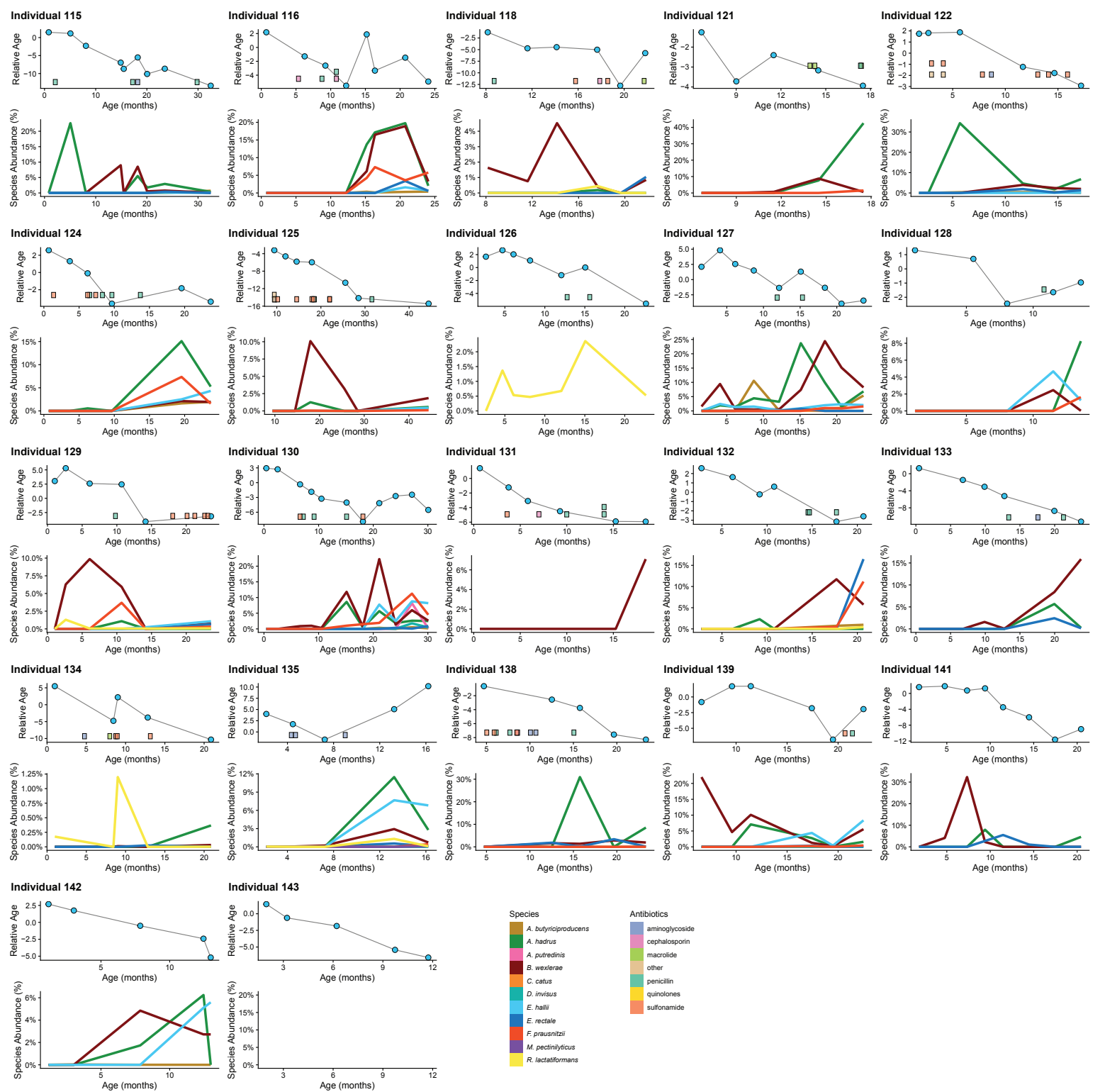

Fig. S6

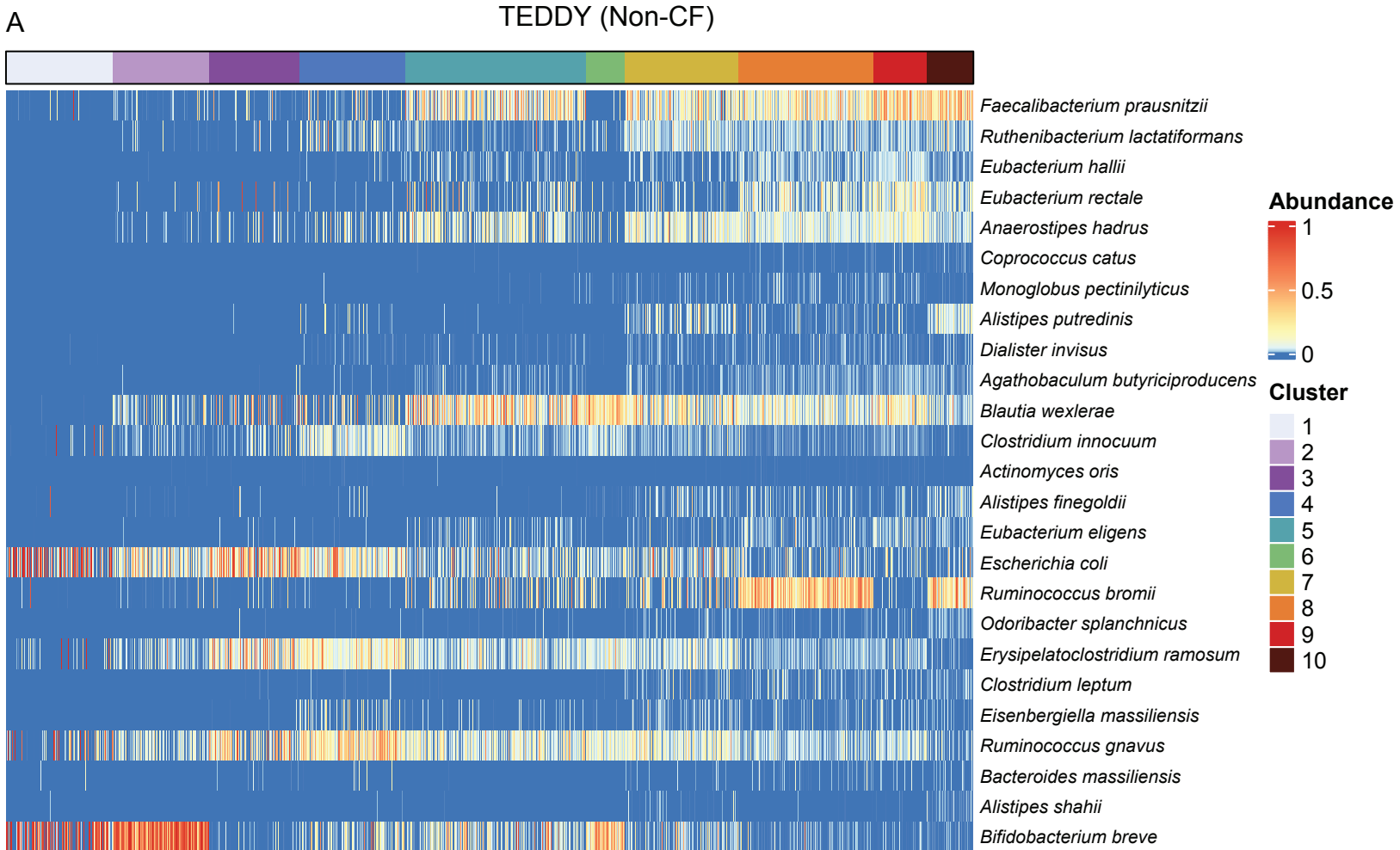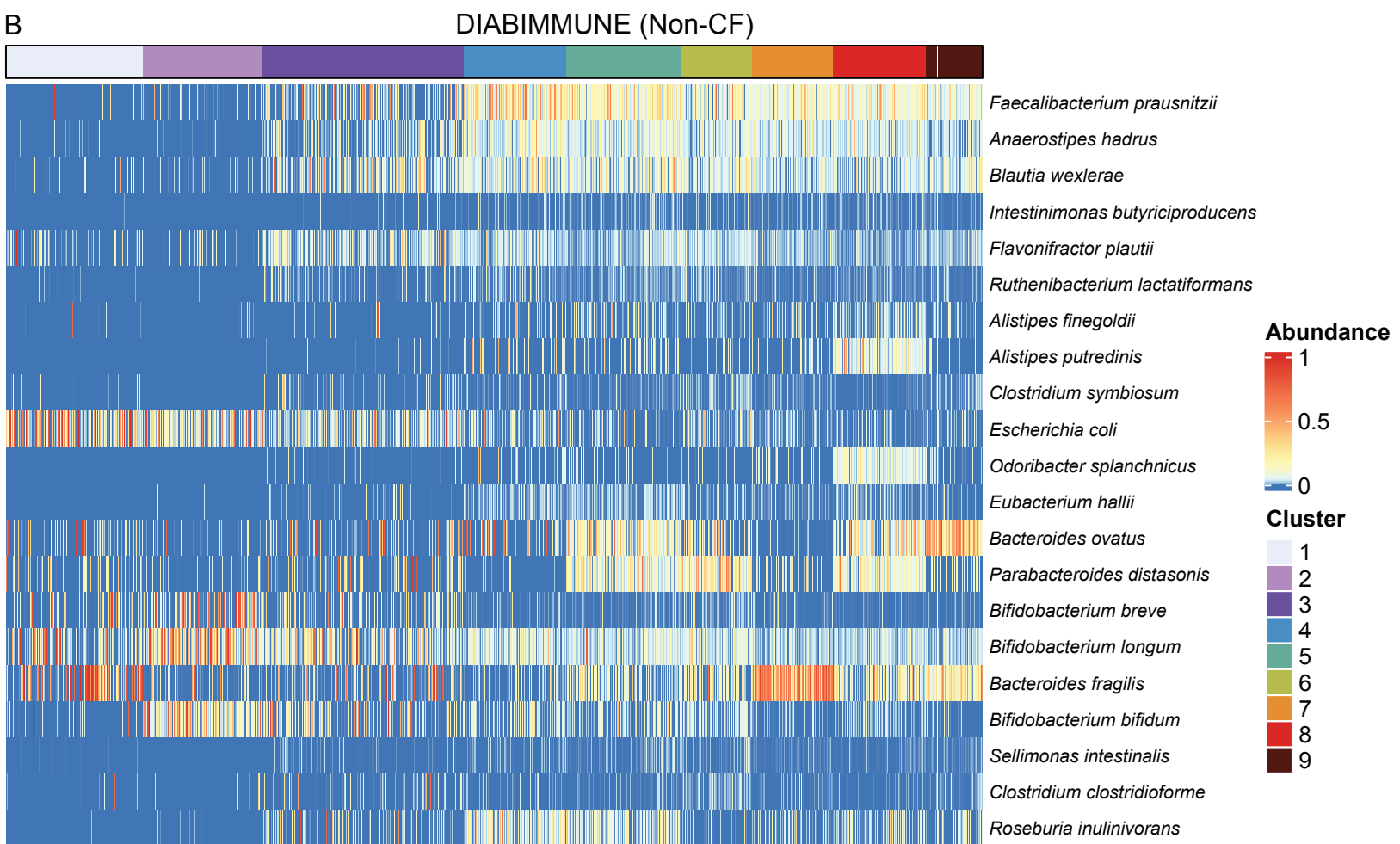

Fig. S7

A

TEDDY Model

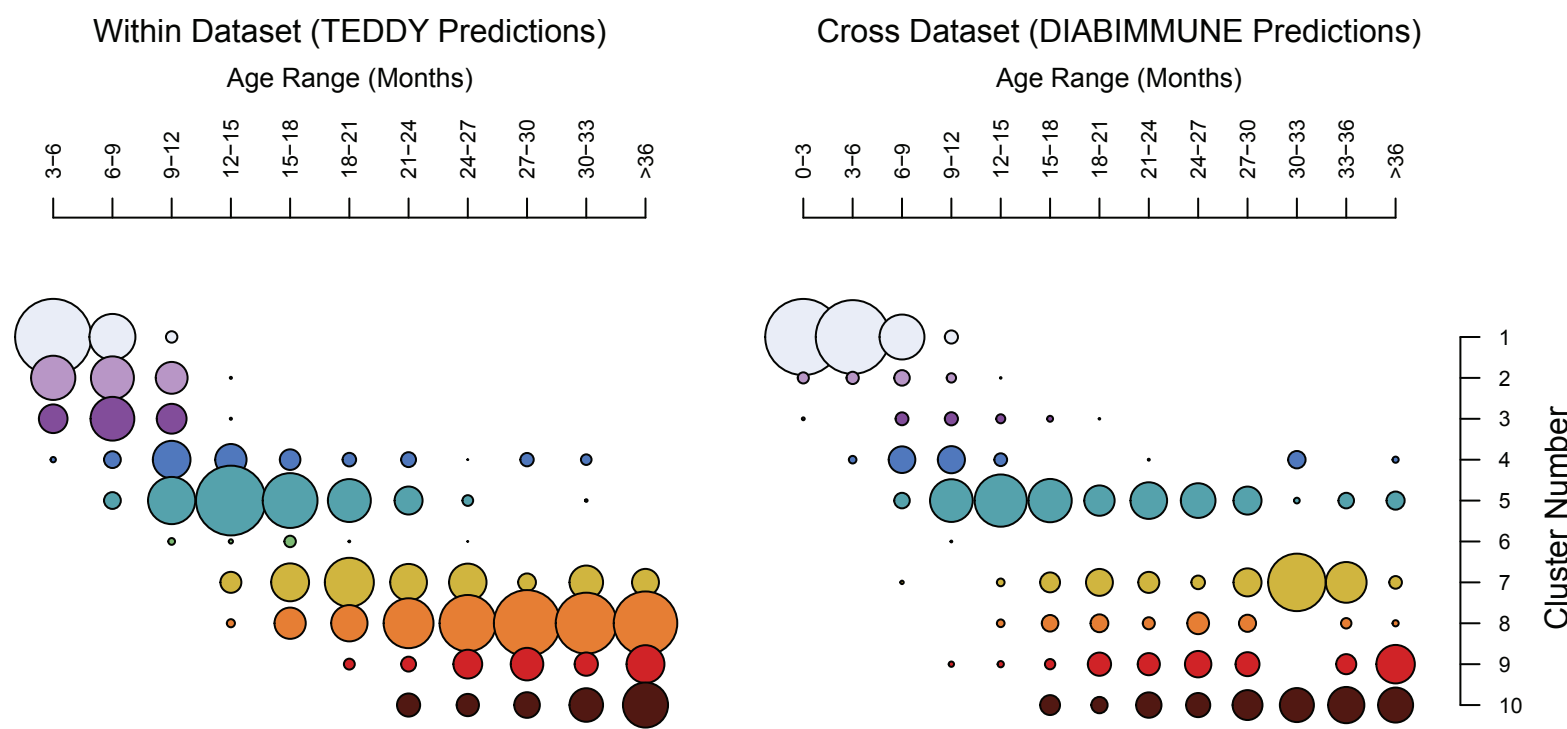

B

DIABIMMUNE Model

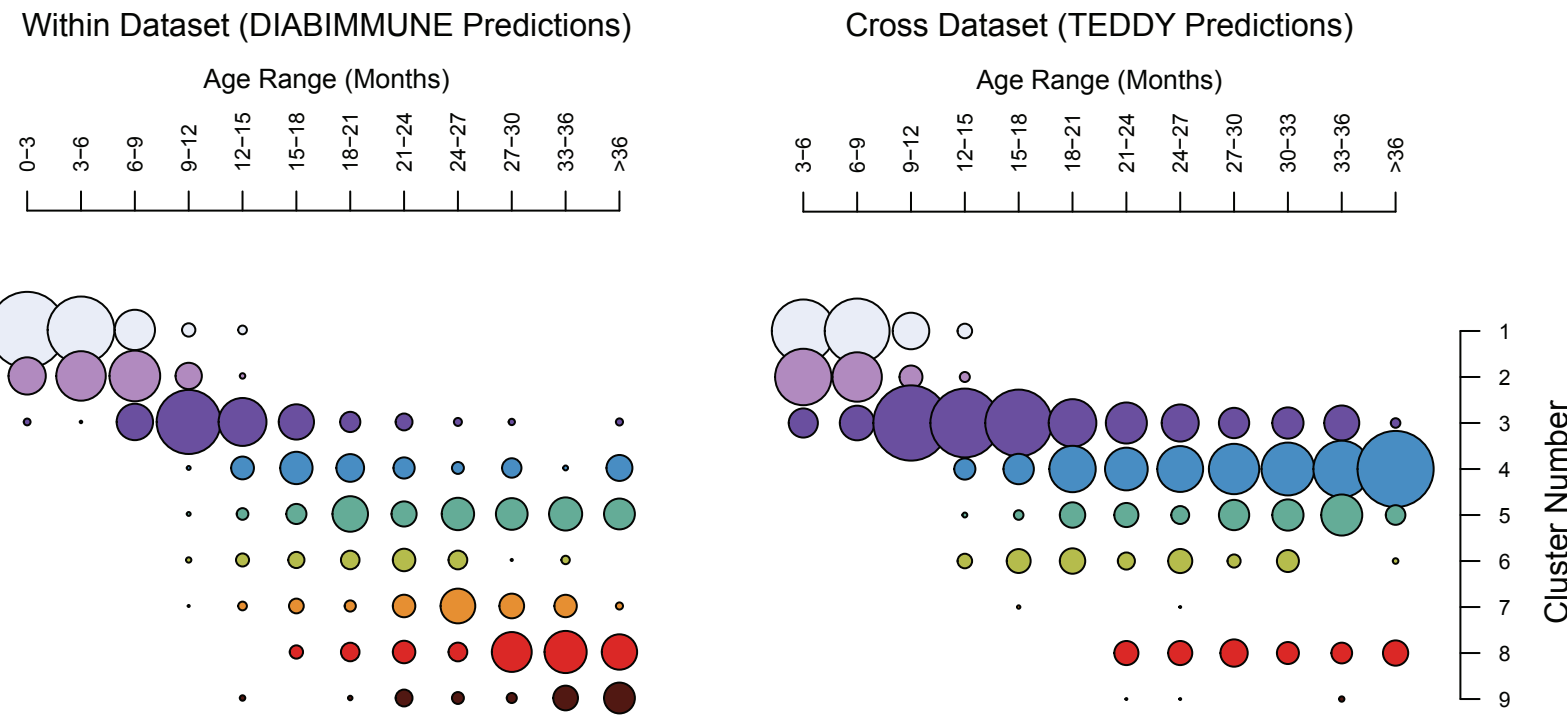

Fig. S8

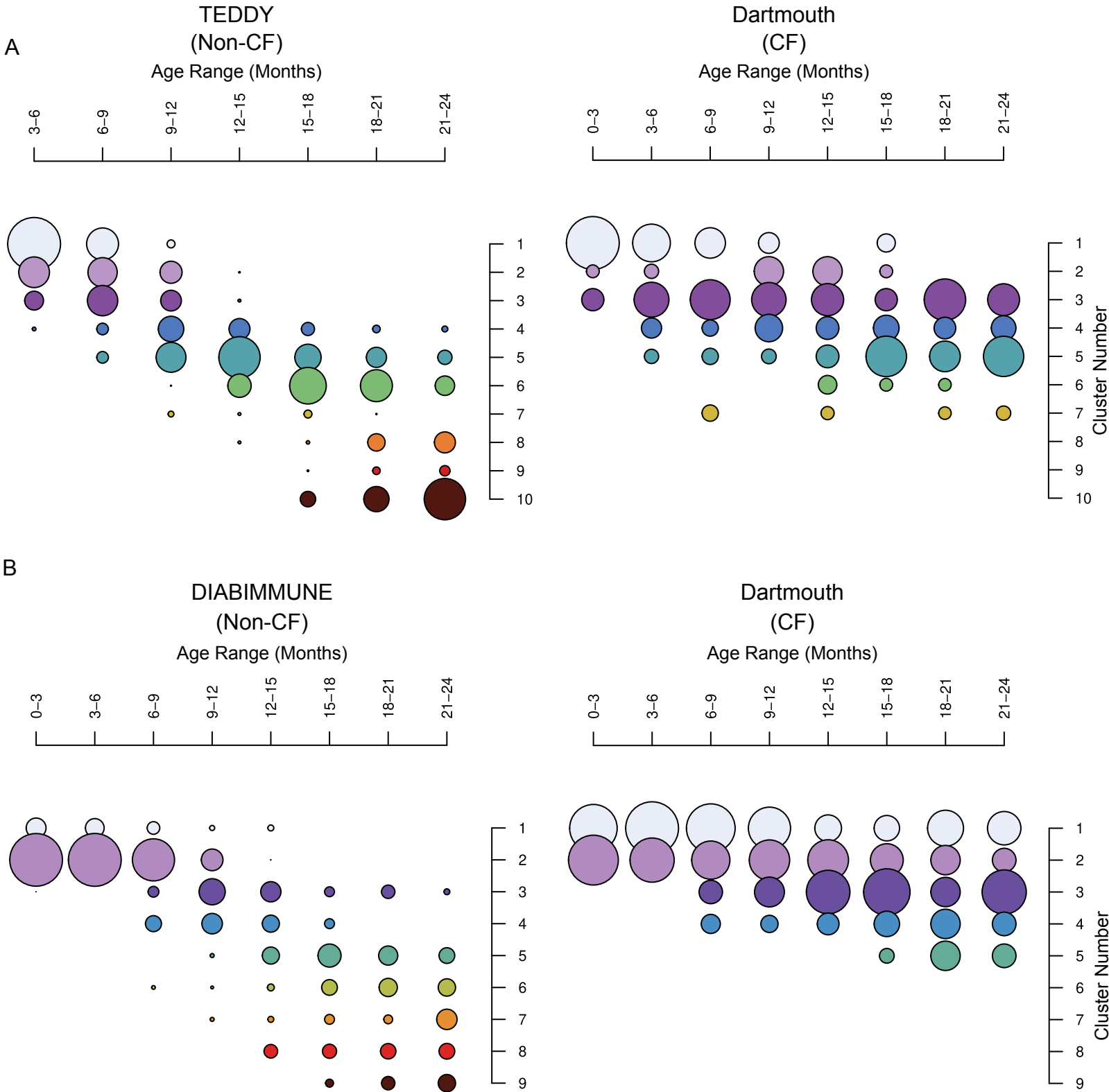

Fig. S9

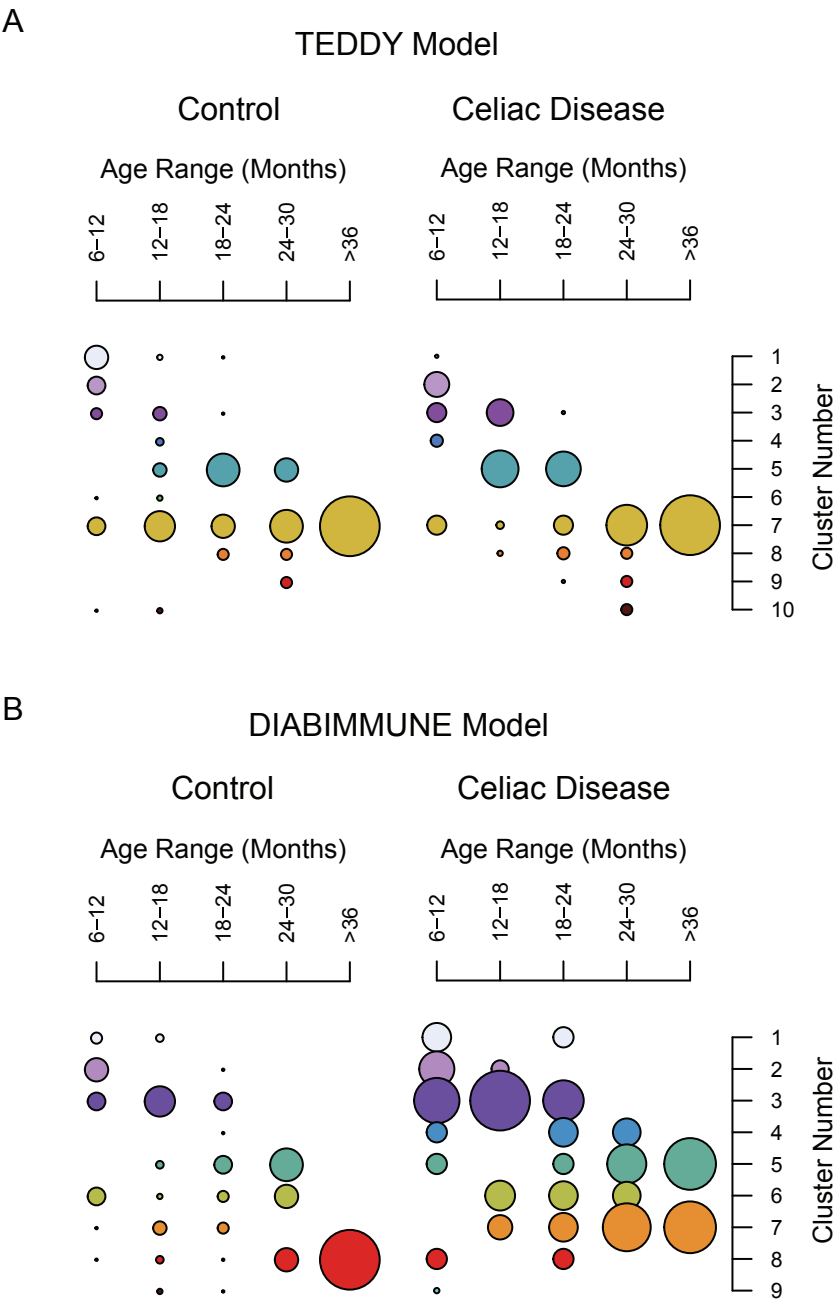

Fig. S10

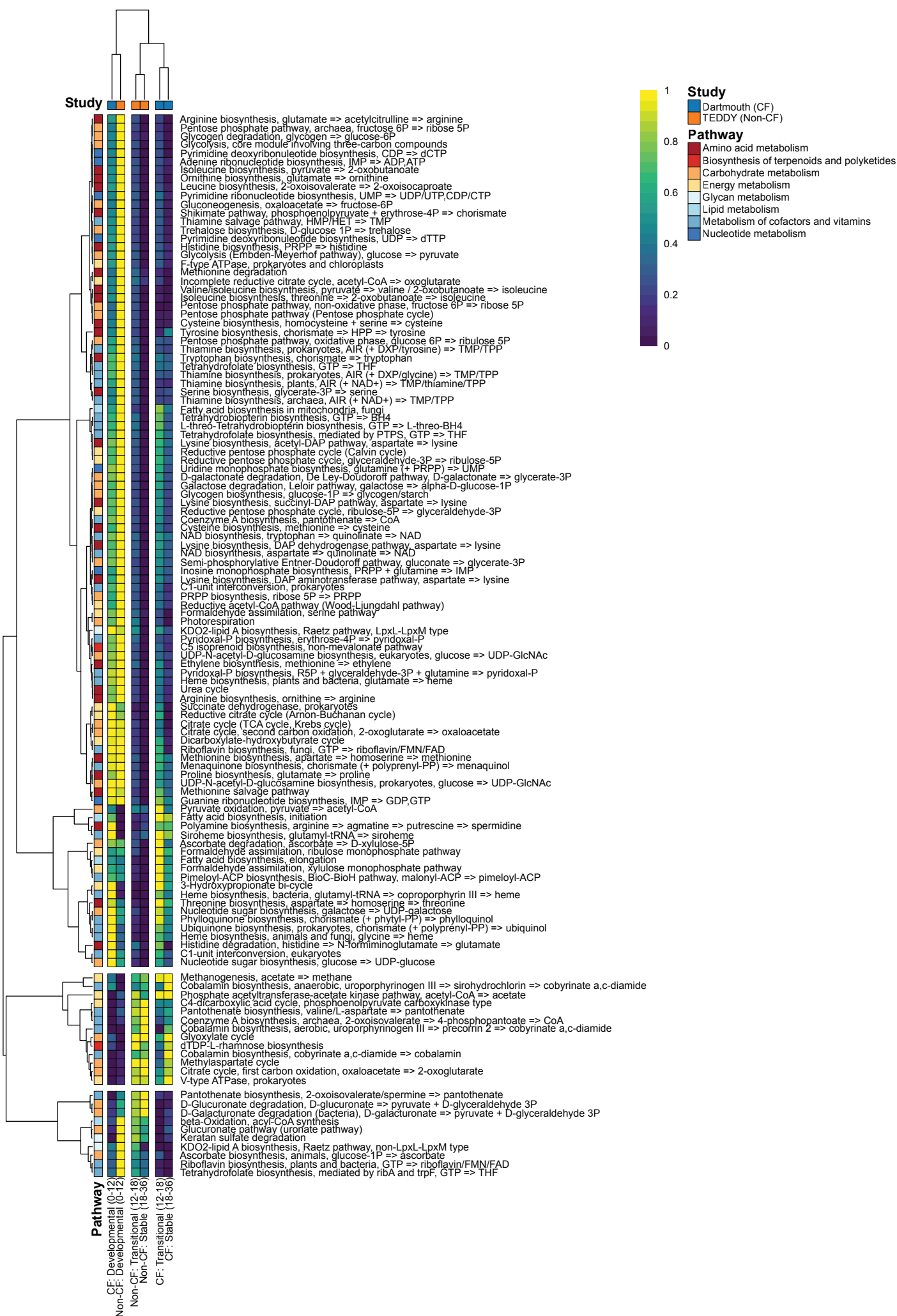

Fig. S11

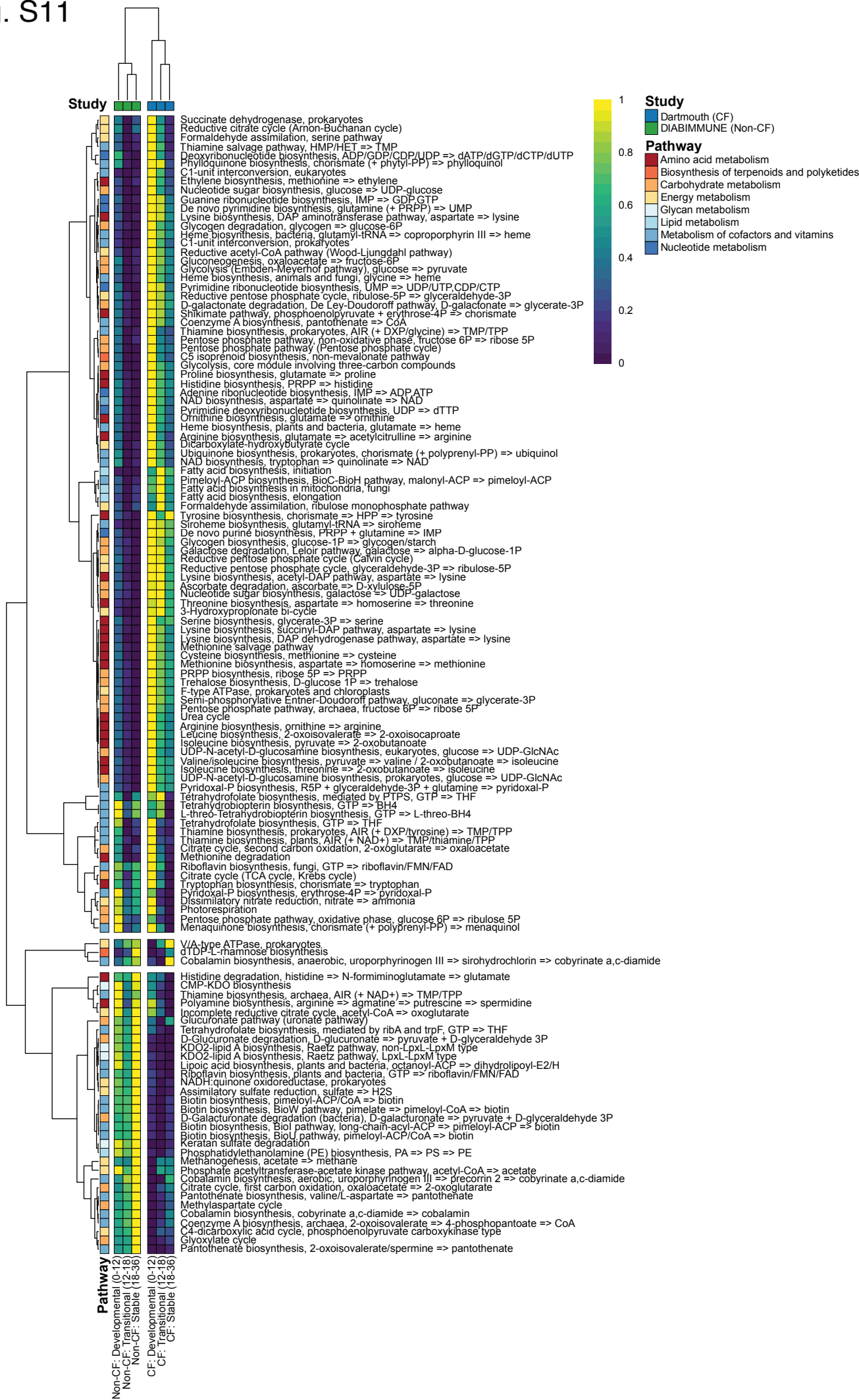

Supplement: Supplemental figures — Figures S4 to S11. [file mbio.03420-24-s0004.pdf]

Fig. S12

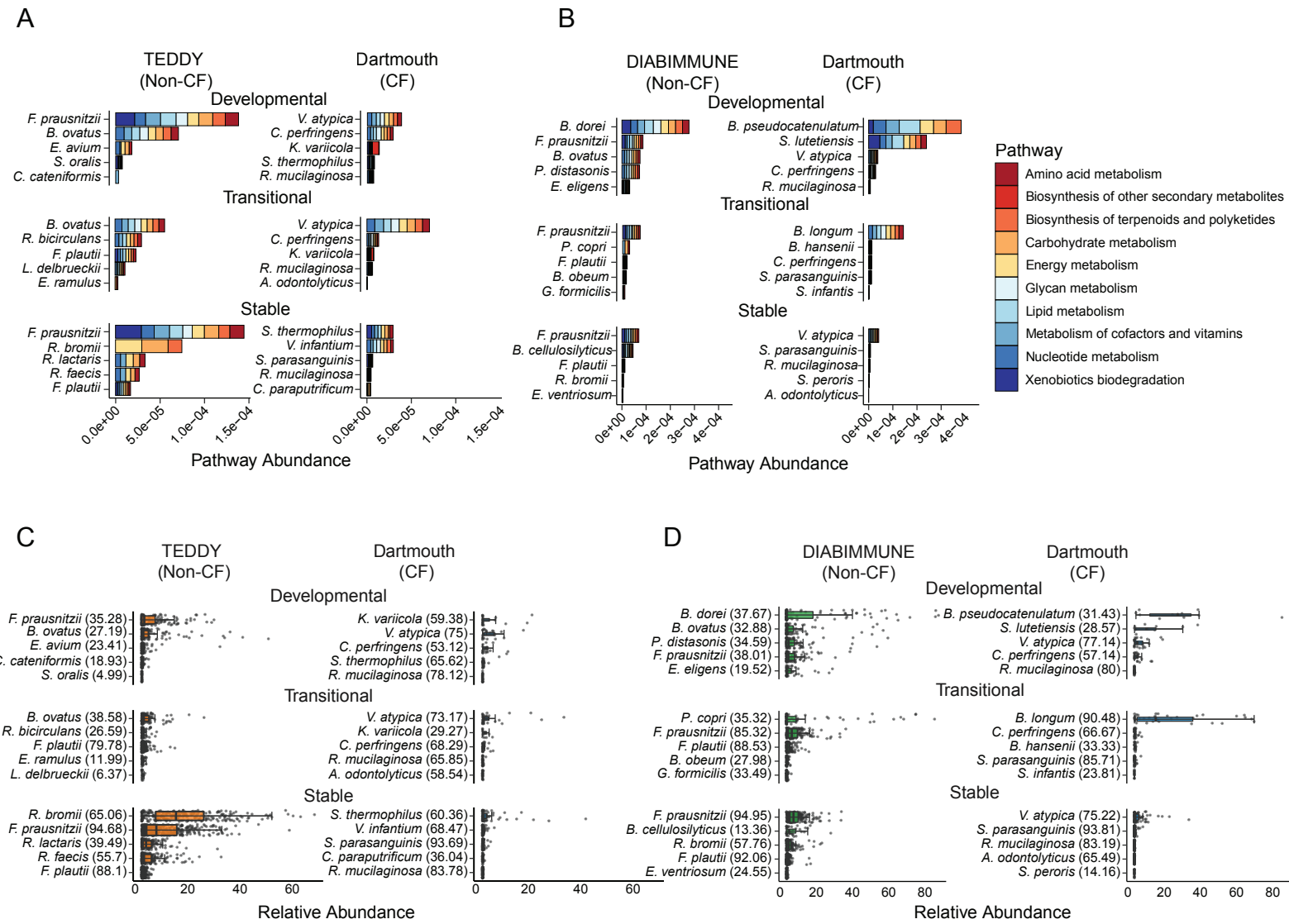

Supplement: Figure S12 — Altered functional capacity in CF compared to non-CF cohorts. [file mbio.03420-24-s0005.pdf]

Fig. S13

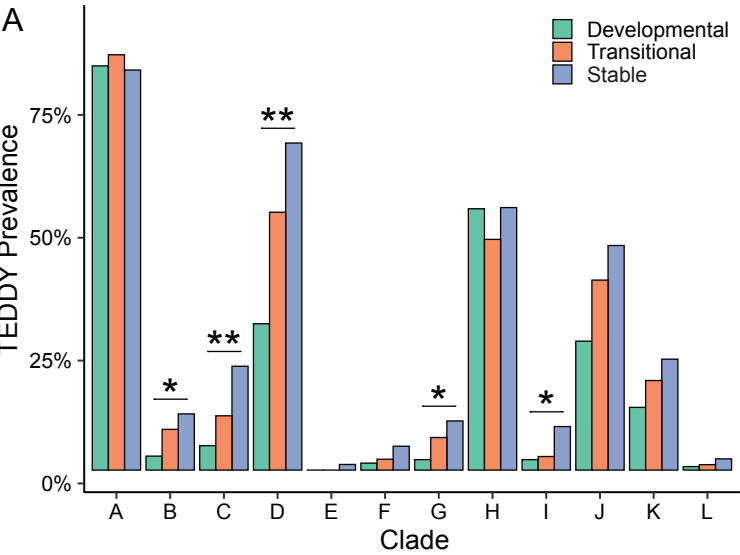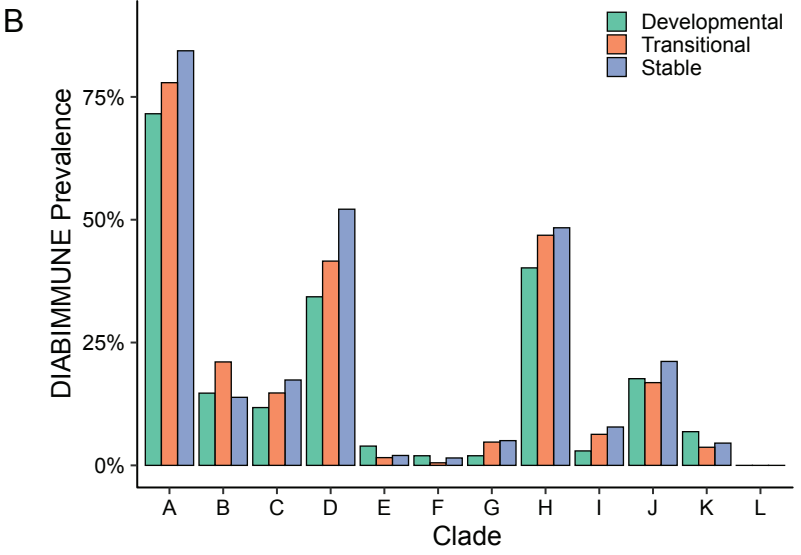

Supplement: Figure S13 — F. prausnitzii increases in prevalence over time in non-CF infants. [file mbio.03420-24-s0006.pdf]
