# Supplementary material for: Persistent delay in maturation of the developing gut microbiota in infants with cystic fibrosis
Source: mBio. 2025 Feb 13;16(3):e03420-24. doi: 10.1128/mbio.03420-24 (PMC11898760; doi:10.1128/mbio.03420-24)
Supplement: Legends — Supplemental figure legends. [file mbio.03420-24-s0007.docx]

**SUPPLEMENTAL FIGURE LEGENDS**

**Fig. S1 | Relative abundance of select phyla differ between infants with CF and non-CF controls.** Select phylum level relative abundance from infants in the Dartmouth (blue), DIABIMMUNE (green), and TEDDY (orange) cohorts over time. Samples were placed into six-month age bins and are shown as boxplots. *P <= 0.05, **P <= 0.01, ***P <= 0.001, ****P <= 0.0001, Wilcoxon ranked sum test (n=2589).

**Fig. S2 | Gut microbiota from CF cohorts exhibit compositional similarity in the first year of life. A**, Phylum level relative abundance of infants in the Dartmouth (left), Hayden CF (right) cohorts from ages 0-12 months. Samples are organized by relative abundance of Proteobacteria. Black dots represent the *E. coli* relative abundance for each sample (n=1255). **B**, PCoA of Dartmouth (blue) and Hayden CF (red) samples from ages 0-12 months (unweighted Unifrac, PERMANOVA). A subset of Hayden samples was randomly selected to match the number of samples in the Dartmouth cohort (n = 101). Vectors indicate the top three species responsible for separation of the two datasets based on Euclidean distance calculations. **C**, PCoA of Dartmouth and Hayden CF samples from ages 0-12 colored by presence of *Bacteroides* species (unweighted UniFrac, PERMANOVA). Samples containing no detectable *Bacteroides* are colored yellow and samples with > 0% relative abundance of *Bacteroides* are colored in blue. Vectors indicate the top three species responsible for separation of the two datasets based on Euclidean distance calculations. **D**, PCoA of Dartmouth (blue), Hayden CF (red), DIABIMMUNE (green), and TEDDY (orange) samples from 6-12 months using unweighted UniFrac calculations. A subset of samples from the Hayden CF, DIABIMMUNE, and TEDDY cohorts were randomly selected to match the number of samples in the Dartmouth group (n = 58). Vectors indicate the top three species (*Escherichia coli*, *Phoecicola vulgatus*, and *Bifidobacterium bifidum*, respectively) responsible for separation of the two datasets based on Euclidean distance calculations.

**Fig. S3 | Altered microbiota compositional dynamics in CF infants. A**, Relative abundance of select bacterial families in Dartmouth (left), DIABIMMUNE (center), and TEDDY (right) cohorts over time. Shaded regions signify 95% confidence intervals (n=2587). **B**, Shannon diversity plot of Dartmouth (blue), DIABIMMUNE (green), and TEDDY (orange) cohorts over time. Each sample is plotted as an individual datapoint, and lines are plotted as averages over time. Shaded regions signify 95% confidence intervals (n=2589). **C**, Shannon diversity values of Dartmouth (blue), DIABIMMUNE (green), and TEDDY (orange) cohorts over time. Samples were binned into 6-month time bins, and statistical tests were run comparing Dartmouth to each of the non-CF control sets. The distribution of data is shown using a boxplot (n=2589). *P <= 0.05, Wilcoxon ranked sum test. **D**, Cross-cohort comparison of Shannon diversity within the first year of life, binned into 0-6 month and 6-12 month time bins. *P <= 0.05, ***P <= 0.001, ****P <= 0.0001, Wilcoxon ranked sum test.

**Fig. S4 | Random forest relative microbiota age model performance evaluated in training sets or cross-dataset. A-B**, Scatter plots show the correlation between the random forest age model predicted age and the actual sample age. Two models were trained, one on non-CF samples from the TEDDY study (**A**, orange, n = 1246) and one on non-CF samples from the DIABIMMUNE study (**B**, green, n = 1154). **C-D**, Scatter plots show the correlation between the random forest age model predicted age and the actual sample age using the best 11 (**C,** orange, n = 1246, TEDDY cohort) or best 10 (**D**, green, n = 1154, DIABIMMUNE cohort) species. **E-F**, Within-dataset predictions were performed on the same training samples in 10-fold cross validation while cross-dataset predictions were done on the other dataset. Value in the upper left of each plot is the spearman correlation between predicted and actual age. **G-H**, Relative microbiota age of Dartmouth cohort when compared to the TEDDY (**G**, n = 190) or DIABIMMUNE (**H**, n = 190) age models using only the top 11 or 10 species. Red dotted line (18 months) signifies the time point at which the slope of the line is no longer significant. The p-values on the plots are a statistical test for a non-zero slope in a linear model between age and relative microbial age. P-value on upper left corner is slope prior to 18 months, p-value on upper right is slope after 18 months.

**Fig. S5 | Microbiota relative age and antibiotic exposure in infants with CF.** Relative age, antibiotic exposure, and age-model species relative abundances are plotted for individuals in the Dartmouth CF infant cohort.

**Fig. S6 | Heatmaps of species relative abundances in non-CF cohorts used in Dirichlet multinomial mixture modeling. A**, Heatmaps of species relative abundance in different clusters derived from the Dirichlet multinomial mixture model. Shown here are all the top 25 species from the random forest age model that were used in the DMM clustering from the TEDDY non-CF cohort. Each column represents a different sample. Heatmap colors represent the relative abundance of each species. The bar at the top of the graph denotes which cluster samples were assigned to and are colored accordingly (n = 1246). **B**, Similar plot with data from the DIABIMMUNE cohort (21 species, n = 1154).

**Fig S7 | DMM cross-validation of non-CF cohorts shows consistent development of microbiome. A**, Dirichlet multinomial mixture clustering (DMMs) of samples from the TEDDY (left), and DIABIMMUNE (right) cohorts using the TEDDY trained model (n = 2400). **B**, DMMs of infant samples from the DIABIMMUNE (left), and TEDDY (right) cohorts using the DIABIMMUNE trained model (n = 2400).

**Fig. S8 | DMM clustering shows delayed development of the CF microbiome compared to non-CF controls in the first two years.** **A**, Dirichlet multinomial mixtures (DMMs) clustering of infants from the TEDDY (left) and Dartmouth (right) cohorts up to 24 months of age. A DMM model was trained on data from TEDDY and applied to cluster data from Dartmouth. Samples were binned into 3-month age bins and clusters were ordered based on the age bin where they are most abundant. Each cluster is denoted by a separate color (n = 1203). **B**, DMMs of infants from the DIABIMMUNE (left) and Dartmouth (right) cohorts plotted in the same way as A (n = 1112).

**Figure S9 | DMM modeling of gut microbiota from infants prior to onset of Celiac Disease. A**, Dirichlet multinomial mixtures (DMMs) of infant samples from the Leonard study using the TEDDY trained model (n = 118). Control samples are found on the left and Celiac Disease (CD) samples are found on the right. Samples were binned into 6-month age bins and colors represent each cluster. **B**, DMMs of infant sample from the Leonard study using the DIABIMMUNE trained model. Leonard control samples are found on the left and Leonard CD samples are found on the right. Samples were binned into 6-month age bins and colors represent each cluster (n = 118).

**Figure S10 | Functional gene abundance is altered in CF infant gut microbiota compared to TEDDY.** Heatmap showing the mean abundance of all significant KEGG modules between the Dartmouth and TEDDY cohorts identified using MaAsLin at each developmental phase. Modules that were found to be significantly different in at least one phase are shown and values were normalized on a per row basis across all time points. The corresponding pathways for each module are also noted. (n=1436).

**Figure S11 | Functional gene abundance is altered in CF infant gut microbiota compared to DIABIMMUNE.** Heatmap showing the mean abundance of all significant KEGG modules between the Dartmouth and DIABIMMUNE cohorts identified using MaAsLin at each developmental phase. Modules that were found to be significantly different in at least one phase are shown and values were normalized on a per row basis across all time points. The corresponding pathways for each module are also noted. (n=1344).

**Figure S12 | Altered functional capacity in CF compared to non-CF cohorts. A-B**, Functional pathway abundances of the top five most statistically significant species at each developmental phase for the Dartmouth and TEDDY (**A**, n=1436) and Dartmouth and DIABIMMUNE (**B**, n=1344) cohorts. Significance was determined by linear mixed effects modeling, and abundances were summed by KEGG pathway. **C-D**, Relative abundances of the top five most statistically significant species at each developmental phase for the Dartmouth and TEDDY (**C**, n=1436) and Dartmouth and DIABIMMUNE (**D**, n=1344) cohorts. Abundance was summed over all individuals for each phase, and the prevalence for each microbe is noted in parentheses.

**Figure S13 | *F. prausnitzii* increases in prevalence over time in non-CF infants. A**, Prevalence of each *F. prausnitzii* clade in the TEDDY cohort, colored by each of the developmental phases. *FDR <= 0.05, **FDR <= 0.01, Fisher’s exact test. P-values were adjusted for multiple corrections using an FDR (n = 1246)**. B**, Prevalence of each *F. prausnitzii* clade in DIABIMMUNE cohort, colored by each of the developmental phases. *FDR <= 0.05, **FDR <= 0.01, Fisher’s exact test. P-values were adjusted for multiple corrections using an FDR (n = 1344).
